# Sex-Specific Cytokine Profiles Associated with Viral Load and Disease Severity in Patients with Severe Fever with Thrombocytopenia Syndrome

**DOI:** 10.3390/microorganisms14102242

**Published:** 2026-10-03

**Authors:** Lianfeng Li, Ling Qiu, Yifei Wang, Qing Dong, Mingzhu Zhang, Peiyu Zhen, Mengying Gao, Shan Lu, Di Zhao, Wenya Tian, Qi Zhou, Luzhao Feng, Lin Zhao, Jie Qian

**Affiliations:** 1 Institute of EcoHealth, School of Public Health, Cheeloo College of Medicine, Shandong University, Jinan 250012, China; 2 School of Population Medicine and Public Health, Chinese Academy of Medical Sciences & Peking Union Medical College, Beijing 100730, China; 3 Key Laboratory of Pathogen Infection Prevention and Control (Peking Union Medical College), Ministry of Education, Beijing 100730, China; 4 Shandong Public Health Clinical Center, Department of Infectious Diseases, Jinan 250013, China; 5 Shandong Provincial Key Laboratory of Intelligent Monitoring, Early Warning, Prevention and Control for Infectious Diseases, Jinan 250014, China

**Keywords:** severe fever with thrombocytopenia syndrome (SFTS), SFTS virus (SFTSV), cytokines, magnitude of cytokine changes, sex differences, viral load, disease severity

## Abstract

Background: Severe fever with thrombocytopenia syndrome (SFTS) is a severe tick-borne viral disease characterized by dysregulated host immune responses. Although previous studies have suggested sex-related differences in immune responses in SFTS, the sex-specific magnitude of cytokine changes and their relationships with viral load and laboratory parameters have not been fully characterized. Methods: We investigated sex differences in cytokine profiles and their associations with viral load and disease severity in 100 confirmed SFTS patients and 60 healthy controls. Nineteen cytokines, viral load, and clinical and laboratory parameters were assessed in acute-phase sera. Correlation analyses were performed to assess the relationships of cytokine changes with viral load and clinical parameters. Multivariable regression analyses were used to evaluate the association between sex and cytokine changes after adjustment for potential confounders, and sex-stratified ROC analyses were conducted to assess the ability of cytokines to discriminate disease severity. Results: Cytokine levels were generally higher in SFTS patients than in controls and increased with disease severity. After normalization to sex-matched controls, females showed higher normalized cytokine ratios for CCL4, IL-17, IL-8, and FGF2, whereas males showed higher normalized ratios for IL-5 and PDGF-AB. Cytokine changes were associated with viral load in both sexes, but these associations did not differ significantly between males and females. After adjustment for potential confounders, FGF2 and IL-17 showed higher normalized cytokine ratios in females, whereas IL-5 and CXCL10 showed higher normalized ratios in males. Exploratory sex-stratified ROC analyses identified several cytokines with relatively high AUCs for discriminating concurrent mild and severe disease, including CXCL10 and CCL4. Conclusions: These findings provide preliminary indications of sex-related differences in cytokine responses during acute SFTS and suggest that the magnitude of cytokine changes may help better characterize sex-specific cytokine responses and their associations with disease severity.

## 1. Introduction

Severe fever with thrombocytopenia syndrome (SFTS) is an emerging tick-borne disease caused by the SFTS virus (SFTSV), which has been officially renamed *Dabie Bandavirus* and is currently classified within the genus Bandavirus, family *Phenuiviridae*, order *Bunyavirales* [1]. The geographic distribution of SFTS is expanding. In addition to widespread prevalence in East Asian countries such as China, South Korea, and Japan, cases have also been reported in other Asian countries, including Vietnam, Myanmar, and Thailand [2,3,4,5]. Serological surveys of SFTSV infection have also been reported in Pakistan and Kenya, Africa [6,7]. Clinically, SFTS presents with diverse manifestations, ranging from mild fever and fatigue to severe thrombocytopenia, bleeding tendency, and multi-organ injury. Some patients may rapidly progress to shock, multi-organ failure, and even death. Despite improvements in diagnostic and therapeutic approaches, the overall case fatality rate remains 7.8% [2], making SFTS a significant emerging infectious disease and public health concern worldwide.

Cytokine storm (CS) is considered an important pathological mechanism underlying the progression of severe SFTS [8]. CS is characterized by excessive release of inflammatory cytokines, aberrant activation of immune cells, and systemic inflammatory responses, which can lead to extensive pathological changes and multi-organ dysfunction, thereby promoting disease progression from mild to severe illness [9,10]. SFTSV infection can induce systemic inflammatory responses accompanied by marked changes in multiple pro-inflammatory and immunoregulatory cytokines, with cytokine responses being closely associated with disease severity and, for some cytokines, viral load [8,11,12]. In addition, cytokine responses in patients with SFTS exhibit sex-related differences [13], suggesting that sex may influence the host immune response to SFTSV infection.

Sex is an important biological determinant of host immune responses. Previous studies have reported sex-related differences in clinical outcomes among patients with SFTS, with male patients showing a higher risk of severe disease and mortality in some cohorts [14,15]. Males and females exhibit differences in immune responses, including cytokine production, lymphocyte activation, and antiviral capacity [16,17]. Similar sex-related differences in host immune responses have also been observed in other viral infections. For example, studies of SARS-CoV-2 infection have reported weaker T-cell responses, dysregulated inflammatory cytokine levels, and more severe organ injury in male patients [18,19]. Although sex-related differences in cytokine levels have been reported in patients with SFTS, potential differences in baseline cytokine levels between healthy males and females may not have been fully accounted for in previous comparisons. Consequently, comparisons based solely on absolute cytokine concentrations may reflect a combination of pre-existing sex-related differences in healthy cytokine levels and disease-associated cytokine alterations. It therefore remains unclear whether the magnitude of cytokine alterations relative to sex-specific healthy reference levels differs between male and female patients with SFTS. In this case–control study, we aimed to characterize sex-related cytokine profiles in patients with SFTS and to evaluate cytokine alterations relative to sex-specific healthy reference levels. We hypothesized that the magnitude of these relative cytokine alterations might differ between male and female patients. After normalization to the median cytokine concentrations of sex-matched healthy controls, we compared cytokine alterations between male and female patients and further examined their associations with viral load, disease severity, and laboratory indicators. Finally, we performed sex-stratified exploratory analyses to examine cytokine features associated with disease severity in male and female patients.

## 2. Materials and Methods

### 2.1. Study Design and Subjects

This study was conducted at the Shandong Public Health Clinical Center between 2023 and 2024. Confirmed cases were defined as patients presenting with acute fever (body temperature ≥ 38 °C) accompanied by thrombocytopenia (platelet count, <100 × 10^9^/L) and/or leukopenia (leukocyte count, <4.0 × 10^9^/L), together with at least one of the following laboratory criteria: (1) successful isolation of SFTSV from clinical specimens; (2) detection of SFTSV RNA by molecular methods; or (3) seroconversion or four-fold increase in antibody titers between acute and convalescent serum samples [20,21].

During the study period, 161 patients with confirmed SFTS were identified. Patients were excluded if they had incomplete clinical or laboratory data, comorbidities, or were admitted >14 days after symptom onset. After applying the predefined exclusion criteria, eligible patients were classified as having mild or severe disease at the time of sample collection according to the Diagnostic and Treatment Protocol for Severe Fever with Thrombocytopenia Syndrome (2023 Edition) [20], independently of the final survival outcome. For the case–control analysis, eligible patients were stratified by sex and disease severity, and the first 25 eligible patients according to medical record number within each stratum were included to ensure balanced representation across the four groups. A total of 100 patients were ultimately included, comprising 25 males with mild disease, 25 males with severe disease, 25 females with mild disease, and 25 females with severe disease. Comparisons between male and female patients were intended to investigate the sex-specific magnitude of cytokine changes and their relationships with viral load and clinical parameters, rather than to estimate sex-specific risks of severe disease or mortality. In parallel, 60 healthy individuals (30 males and 30 females) were recruited as controls through a local community-based health screening program. The inclusion criteria were negative SFTSV RNA testing in serum/blood samples and negative SFTSV IgG or IgM antibody testing in serum. Individuals with other infections, chronic diseases, or symptoms similar to those of SFTS were excluded.

### 2.2. Sample Collection

Serum samples were collected from SFTS patients during hospitalization in the acute phase of illness. Blood samples were collected on the day of admission or the following day. Clinical and epidemiological data were collected, including demographic characteristics, medical history, symptoms and signs, laboratory test results, and clinical outcomes. All relevant information was independently extracted from electronic medical records by two investigators and cross-checked to ensure data accuracy. In addition, serum samples were collected from healthy controls, and basic demographic information was recorded. All serum samples were stored at −80 °C until analysis.

### 2.3. Viral RNA Extraction and PCR Detection

Viral RNA was extracted from acute-phase serum samples of SFTS patients using the QIAamp Viral RNA Kit (Qiagen, 52904, Hilden, Germany) according to the manufacturer’s instructions. The extracted RNA was subsequently used for RT-qPCR using the One Step PrimeScript™ RT-PCR Kit (Takara, RR064A, Shiga, Japan) following the manufacturer’s instructions. SFTSV RNA was detected and quantified using S segment-specific primers. The primer sequences and amplification conditions were adopted from previously published methods [22]. The resulting RT-qPCR measurements were used to determine the serum viral load of SFTSV during the acute phase of infection.

### 2.4. Serum Cytokine Measurement

Serum cytokine levels were quantitatively measured using the Luminex^®^ Discovery Assay Human Premixed Multi-Analyte Kit (R&D Systems, Minneapolis, MN, USA). A total of 19 cytokines were analyzed, including tumor necrosis factor alpha (TNF-α), interleukin-6 (IL-6), interleukin-8 (IL-8), interferon gamma-induced protein 10 (CXCL10), interferon gamma-induced protein 9 (CXCL9), monocyte chemoattractant protein-1 (CCL2), macrophage inflammatory protein-1β (CCL4), Eotaxin (CCL11), interleukin-2 (IL-2), interleukin-4 (IL-4), interleukin-5 (IL-5), interleukin-7 (IL-7), interleukin-17 (IL-17), interferon gamma (IFN-γ), interleukin-1 receptor antagonist (IL-1RA), platelet-derived growth factor (PDGF-AB), basic fibroblast growth factor (FGF2), granulocyte–macrophage colony-stimulating factor (GM-CSF), and granulocyte colony-stimulating factor (G-CSF). Serum cytokine concentrations were measured using a Luminex-based multiplex assay according to the manufacturer’s instructions. All serum samples were processed under the recommended conditions, and quantitative measurements were performed using the Luminex 200 system. Cytokine concentrations were determined using standard curves according to the manufacturer’s instructions. For measurements reported as outside the measurement range, the corresponding boundary values indicated in the assay results were used for analysis; values below the lower boundary were assigned the lower boundary value, whereas values above the upper boundary were assigned the upper boundary value. All samples were measured in triplicate within the same experimental batch.

### 2.5. Statistical Analysis

The Shapiro–Wilk test was used to assess the normality of continuous variables. Data with a normal distribution are presented as means and standard deviations (SDs); comparisons between two groups were performed using the independent-samples Student’s *t* test. Non-normally distributed continuous variables are presented as median with interquartile range; comparisons between two groups were performed using the Mann–Whitney *U* test. Categorical variables are expressed as counts and percentages and were compared using the chi-square (χ^2^) test or Fisher’s exact test, as appropriate. Correlations between variables were assessed using Spearman correlation coefficients. Cytokine concentrations were log10-transformed before PCA and hierarchical clustering. PCA was performed using the FactoMineR package in R (version 4.4.2) with mean centering and unit-variance scaling. Hierarchical clustering of cytokines was performed using Euclidean distance and complete linkage. For analyses involving multiple cytokine comparisons, hypotheses addressing the same analytical question were grouped into separate comparison families, and *p* values were adjusted using the Benjamini–Hochberg (BH) false discovery rate (FDR) procedure within each comparison family. BH-adjusted *p* < 0.05 was considered statistically significant.

### 2.6. Multiple Linear Regression Analysis

To evaluate the association between sex and normalized cytokine levels, multiple linear regression models were constructed for nine cytokines (CCL4, FGF2, IL-17, IL-8, CXCL10, IL-5, PDGF-AB, IL-1RA, and CCL11), with sex as the primary independent variable. Age, disease severity (mild vs. severe), viral load, time interval from symptom onset to sample collection, and laboratory parameters (AST, ALT, LDH, CK, platelet count, and white blood cell count) were included as covariates. Multicollinearity was assessed using variance inflation factors (VIFs). Model diagnostics were performed to assess linearity, residual distributions, heteroscedasticity, and potentially influential observations. Heteroscedasticity-robust HC3 standard errors were used for statistical inference. Sensitivity analyses were performed using parsimonious models including sex, age, time from symptom onset to sampling, viral load, and disease severity. Sex × severity and sex × viral load interaction terms were additionally examined. Regression coefficients (β) and 95% confidence intervals (CIs) were used to assess the strength and direction of associations. *p* values were adjusted using the Benjamini–Hochberg (BH) procedure.

### 2.7. Sex-Stratified ROC Analysis

Based on the preceding cytokine profiling analysis, nine cytokines (CCL4, FGF2, IL-17, IL-8, CXCL10, IL-5, PDGF-AB, IL-1RA, and CCL11) were included in exploratory sex-stratified ROC analyses of concurrent disease severity discrimination in patients with SFTS. ROC analyses were performed separately in male and female patients using the full sex-specific samples.

ROC curve analyses were performed for each of the nine cytokines within each sex. The area under the ROC curve (AUC) was used to assess the discriminatory ability of each cytokine to distinguish between mild and severe SFTS. The optimal cutoff value was determined using the Youden index, and the corresponding sensitivity and specificity were calculated. Bootstrap resampling was used to obtain 95% CIs for the ROC estimates. For graphical clarity, only ROC curves with an AUC ≥ 0.75 were displayed; this threshold was used solely for visualization and was not used for cytokine selection or determination of statistical significance. The ROC analyses were considered exploratory assessments of concurrent severity discrimination and were not interpreted as predictive performance or as evidence of differences in ROC performance between males and females. All statistical analyses were performed in R, with the pROC package primarily used for ROC analyses.

## 3. Results

### 3.1. Demographic, Clinical, and Laboratory Characteristics of the Study Population

The demographic, clinical, and laboratory characteristics of the study population are summarized in Supplementary Table S1. The patient screening and selection process is shown in Figure S1. There was no significant difference in age between male and female patients with mild disease (67 [56–75] vs. 60 [53–69] years, *p* = 0.174). In the severe group, male patients were significantly older than female patients (70 [68–74] vs. 66 [58–72] years, *p* = 0.016). Overall, male patients were also significantly older than female patients (69 [65–75] vs. 64 [54–70] years, *p* = 0.008) (Table S1). Results showed that among mild cases, no significant differences were observed between male and female patients in other clinical characteristics or laboratory test results; among severe cases, except for age, no significant differences were observed between sexes in other clinical or laboratory parameters. Overall comparisons indicated that male patients had significantly higher creatine kinase (CK) levels than female patients (*p* = 0.02). In addition, the median age was 69 years (IQR, 65–75) in male patients and 66 years (IQR, 65–71) in male healthy controls, with no statistically significant difference in age distribution (*p* = 0.170). Similarly, the median age was 64 years (IQR, 54–70) in female patients and 61 years (IQR, 56–67) in female healthy controls, with no statistically significant difference (*p* = 0.578) (Table S2).

### 3.2. Cytokine Expression Was Higher in Severe SFTS Patients than in Mild Cases

**Figure 1:**
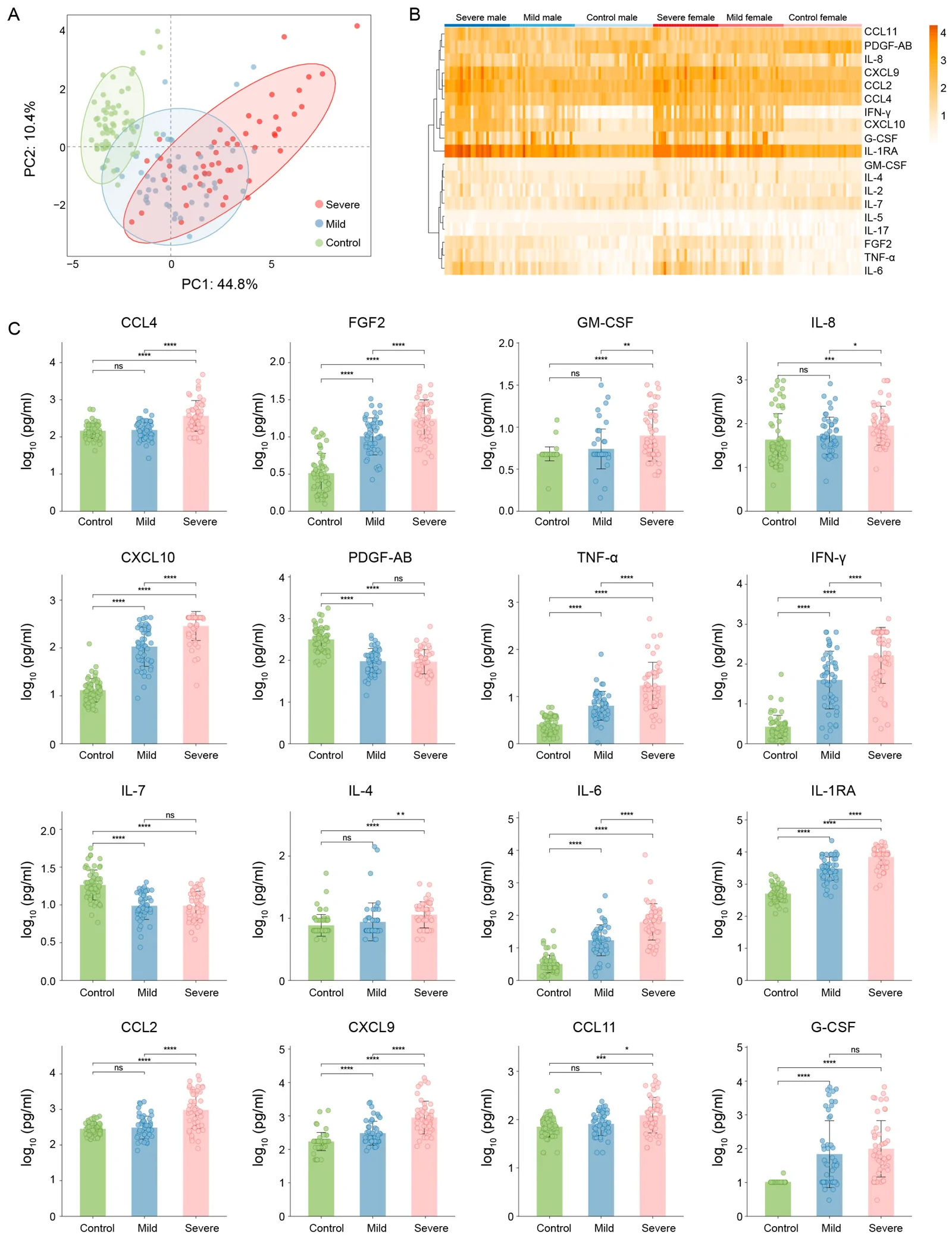
Cytokine expression profiles in SFTS patients and healthy controls. (**A**) Principal component analysis (PCA) based on all measured cytokines in controls, mild cases, and severe cases. (**B**) Expression levels of 19 cytokines across different groups after log_10_ transformation. Color intensity from light to dark indicates low to high cytokine expression levels. (**C**) Comparison of cytokine levels among control, mild, and severe groups. For visualization purposes, data are presented as mean ± SD, with each dot representing an individual sample. Pairwise comparisons between groups were performed using the Mann–Whitney *U* test with BH correction for multiple comparisons. * *p* < 0.05, ** *p* < 0.01, *** *p* < 0.001, **** *p* < 0.0001; ns, not significant.

To systematically assess the immune response characteristics in SFTS patients, 19 cytokines related to immune function were quantified using the Luminex 200 system protein array. Principal component analysis (PCA) revealed a clear separation between severe patients and healthy controls (Figure 1A). Mild patients were distributed between healthy controls and severe patients; although there was some overlap with both groups, they still formed a relatively distinct cluster, suggesting that patients with different disease severities exhibit differences in overall immune response. At the same time, mild and severe cases showed relatively close distributions in the principal component space, indicating that they share similar immune response profiles. Hierarchical clustering heatmap analysis divided the 19 cytokines into two expression clusters (Figure 1B). One cluster of cytokines exhibited overall higher expression levels, including IL-1RA, G-CSF, CXCL10, IFN-γ, CCL4, CCL2, CXCL9, IL-8, PDGF-AB, and CCL11; the other cluster showed relatively lower expression levels, including IL-6, TNF-α, FGF2, IL-17, IL-5, IL-2, IL-4, IL-7 and GM-CSF. Regardless of the cluster, expression levels were generally higher in patients than controls, with severe patients showing higher levels than mild cases.

Among the 19 cytokines measured, 16 showed significant differences in SFTS patients with mild and/or severe disease compared to healthy controls (Figure 1C). Specifically, seven cytokines (TNF-α, IL-6, CXCL10, IFN-γ, IL-1RA, FGF2, and CXCL9) were elevated in both mild and severe patients relative to healthy controls, with levels significantly higher in severe cases than in mild cases (Figure 1C). IL-7 and PDGF-AB were lower in both mild and severe patients compared to healthy controls (Figure 1C). Three cytokines (IL-17, IL-5, and IL-2) showed no significant differences between mild and severe patients (Figure S2). In addition, CCL4, IL-4, and CCL11 were significantly elevated only in severe patients but not in mild patients, showing specific differences compared with healthy controls (Figure 1C).

### 3.3. Significant Sex Differences in Cytokine Expression Were Observed in Both Mild and Severe SFTS Patients

**Figure 2:**
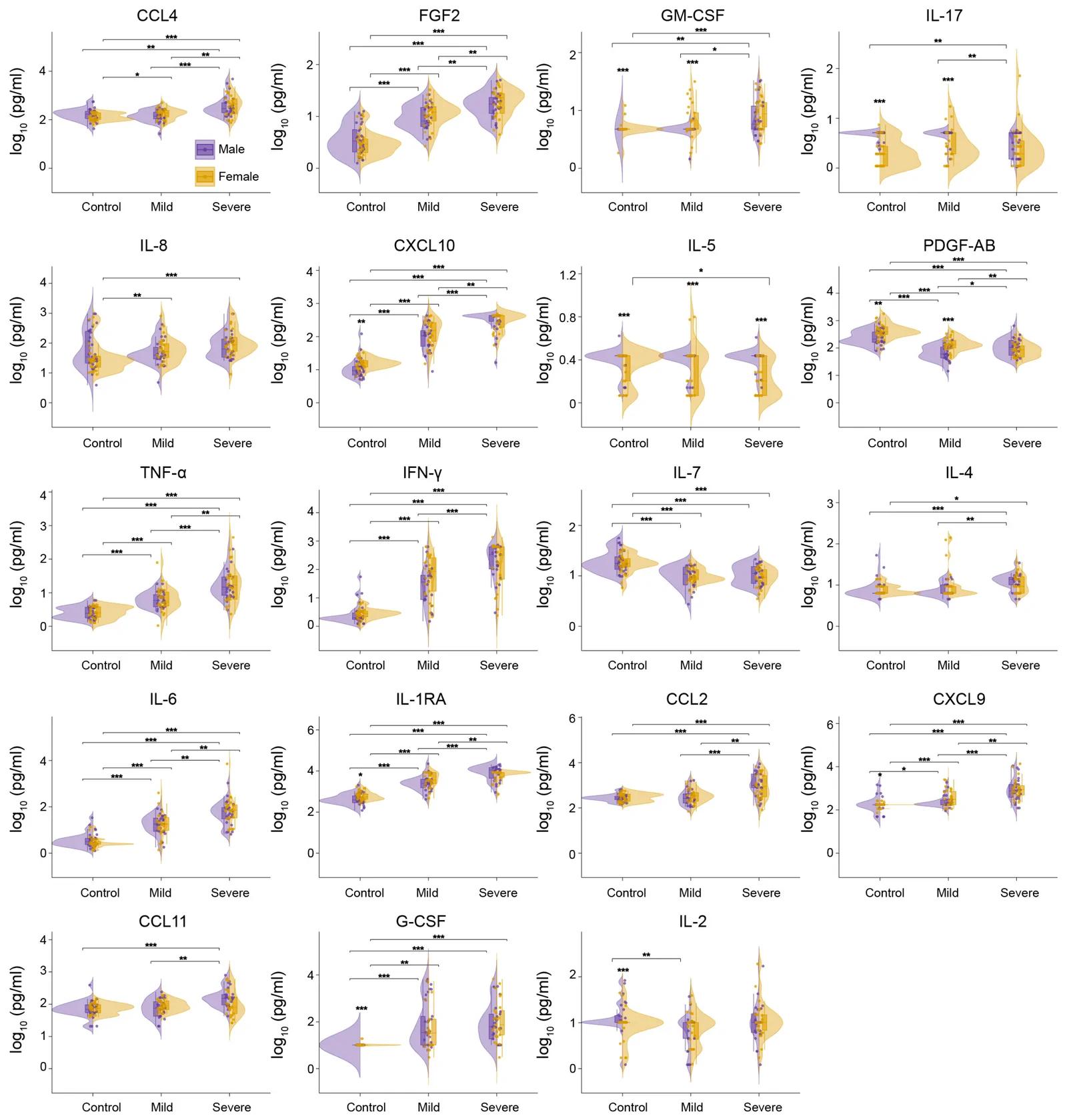
Comparison of cytokine levels by sex and disease severity. Expression levels of 19 cytokines were log_10_ transformed. Pairwise comparisons were performed using the Mann–Whitney *U* test, with *p* values adjusted using the Benjamini–Hochberg (BH) method for multiple comparisons. In box plots, the central line indicates the median; the upper and lower edges of the box represent the third (Q3) and first (Q1) quartiles, respectively; whiskers extend to 1.5 times the interquartile range. * *p* < 0.05, ** *p* < 0.01, *** *p* < 0.001.

We further compared cytokine expression between males and females within each disease severity group. In healthy controls, GM-CSF, IL-17, CXCL10, IL-5, PDGF-AB, IL-1RA, CXCL9, G-CSF and IL-2 showed significant sex differences. In mild patients, GM-CSF, IL-17, IL-5 and PDGF-AB exhibited significant sex differences, whereas in severe patients, only IL-5 differed significantly between sexes (Figure 2). These results suggest that some of the observed sex-related differences may not be entirely attributable to the disease itself, but rather reflect baseline differences in cytokine levels between males and females, which could partially influence comparisons of absolute expression levels under disease conditions.

To further clarify the true changes in cytokine levels during disease progression, we compared male mild and severe patients with male healthy controls, and female mild and severe patients with female healthy controls. The results showed that for most cytokines, expression levels were higher in severe patients than in mild patients, exhibiting a consistent trend across both sexes (Figure 2). Therefore, simply comparing absolute cytokine levels between males and females may not adequately reflect immune response changes associated with disease progression.

### 3.4. Sex-Specific Differences in Normalized Cytokine Ratios Relative to Healthy Reference Levels

**Figure 3:**
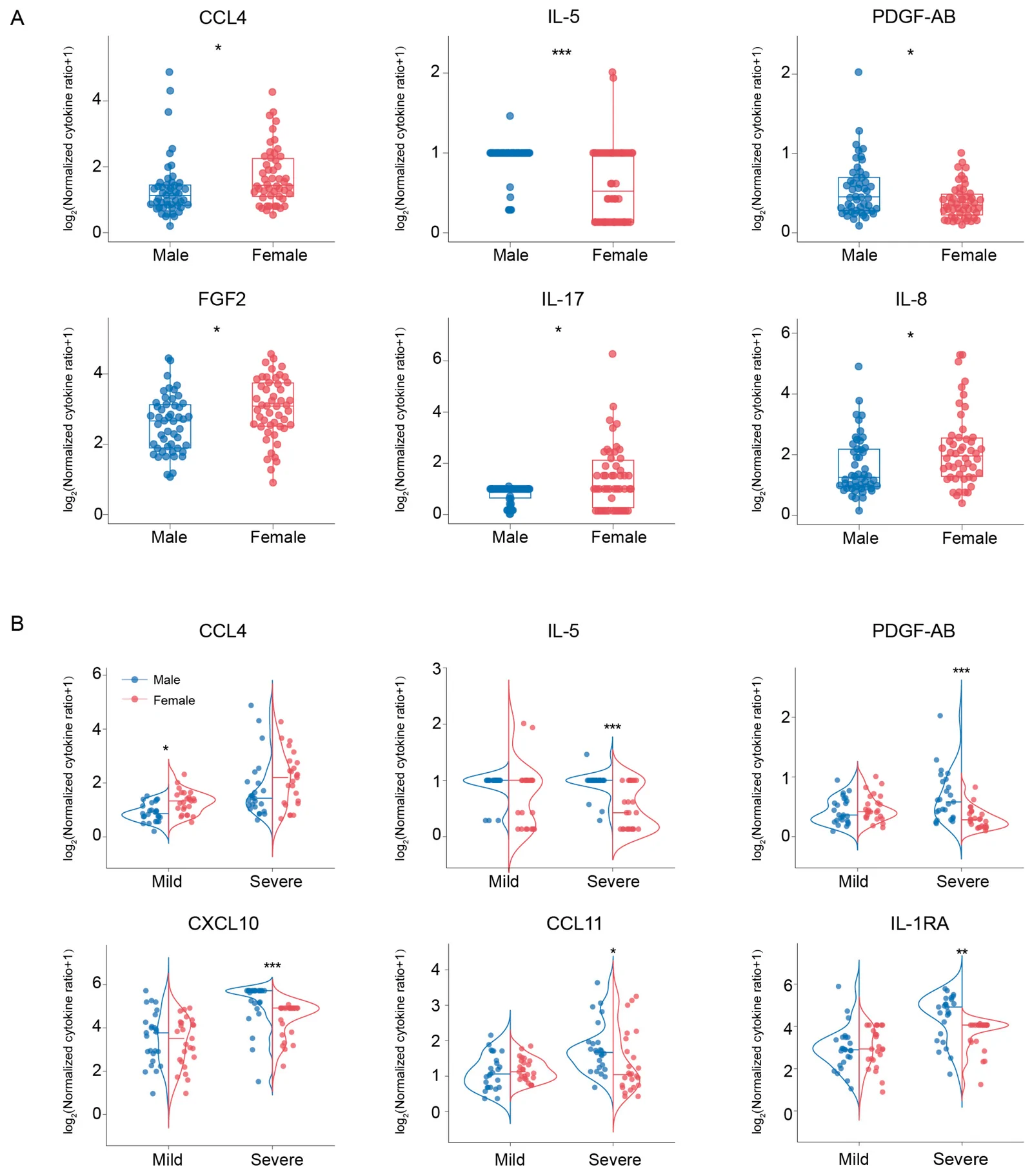
Sex-specific differences in normalized cytokine ratios relative to healthy reference levels. Normalized cytokine ratios were log_2_-transformed and calculated as log_2_[(individual value/median of the control group) + 1] for analysis and visualization. (**A**) Comparison of normalized cytokine ratios between males and females. (**B**) Comparison of normalized cytokine ratios between males and females stratified by disease severity. Statistical comparisons in (**A**,**B**) were performed using the Mann–Whitney *U* test with Benjamini–Hochberg (BH) correction for multiple comparisons. * *p* < 0.05, ** *p* < 0.01, *** *p* < 0.001.

To account for sex-related differences in cytokine levels among healthy individuals, we normalized each patient’s cytokine concentration to the median concentration of the corresponding sex-matched healthy-control group and expressed the result as a log_2_-transformed normalized cytokine ratio log_2_[(measured value/median of control) + 1]. CCL4, FGF2, IL-17, and IL-8 showed higher normalized ratios in female patients compared with males (BH-adjusted *p* < 0.05). In contrast, IL-5 and PDGF-AB exhibited significantly higher normalized ratios in male patients than in females (BH-adjusted *p* < 0.05) (Figure 3A, Table S3). Further stratified analysis by disease severity revealed that the normalized ratio of CCL4 exhibited a significant sex difference in mild cases. CXCL10, IL-5, PDGF-AB, CCL11 and IL-1RA displayed significantly higher normalized ratios in males than in females among severe patients (BH-adjusted *p* < 0.05) (Figure 3B, Table S4). The remaining cytokines did not show statistically significant sex differences after BH correction in the corresponding analyses (Figures S2 and S3, Tables S3 and S4). To directly assess whether the association between disease severity and normalized cytokine ratios differed by sex, sex × disease severity interactions were further tested for all 19 cytokines. After BH correction, only PDGF-AB showed a significant sex × disease severity interaction (interaction estimate = 0.409, 95% CI: 0.186–0.632, *p* < 0.001, BH-adjusted *p* = 0.008). No other cytokine showed a significant interaction after BH correction (Table S5).

### 3.5. Associations of Cytokine Changes with Viral Load and Disease Severity

**Figure 4:**
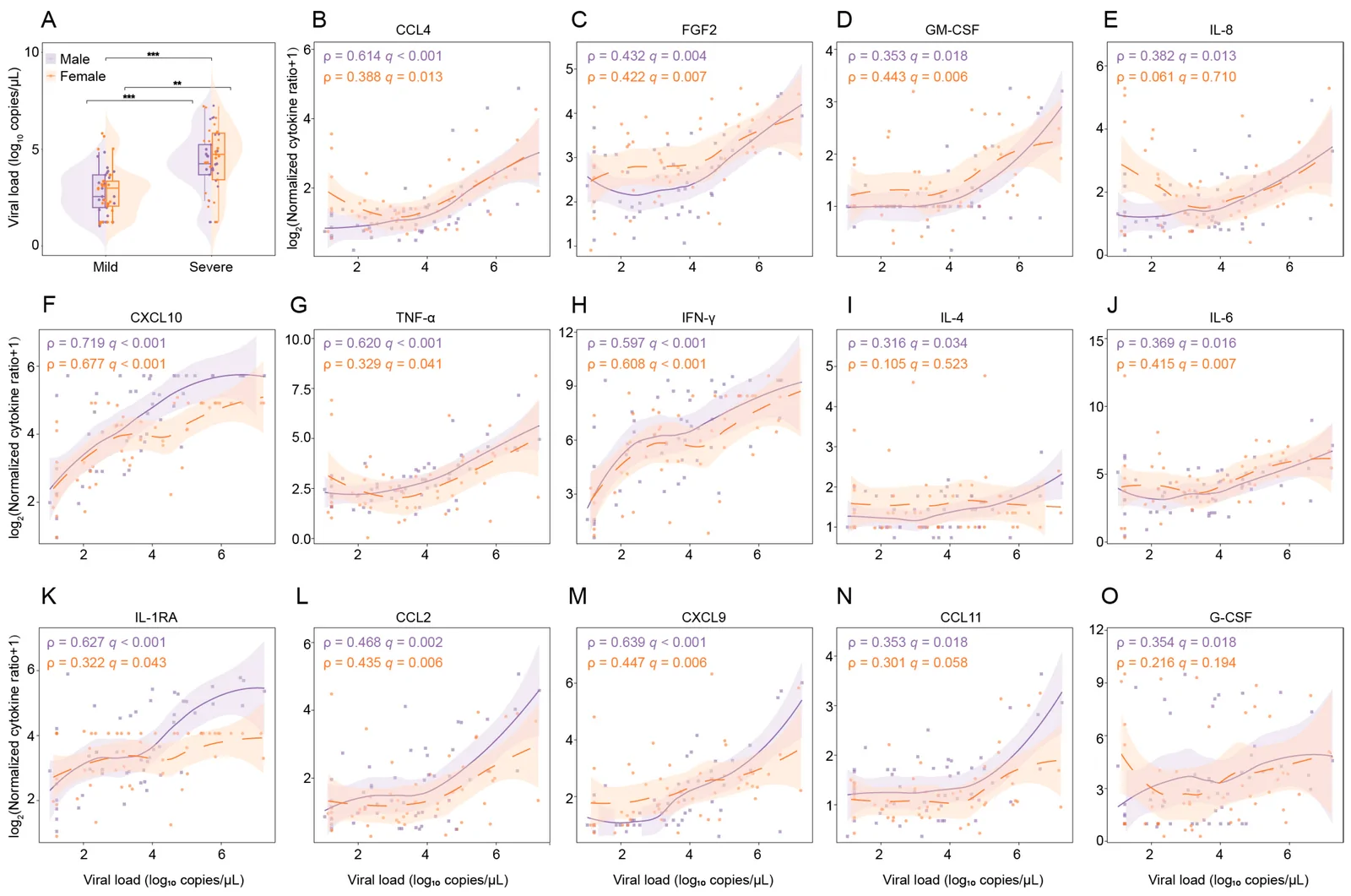
Associations between viral load and normalized cytokine ratios. Normalized cytokine ratios were log_2_-transformed and were calculated as log_2_[(individual value/median of the control group) + 1] for analysis and visualization. (**A**) Comparisons of viral load between mild and severe cases in male and female patients separately, as well as in the overall cohort. Viral load data were log_10_ transformed. (**B**–**O**) Spearman correlation analyses were performed separately in male and female patients to assess the associations between viral load and normalized cytokine ratios. *q* values represent *p* values adjusted using the Benjamini–Hochberg (BH) method. ** *p* < 0.01, *** *p* < 0.001.

We quantitatively measured viral load in all enrolled patients and found no significant differences between males and females, in either mild or severe cases. However, viral loads in both male and female severe patients were significantly higher than those in their respective mild cases (both *p* < 0.01) (Figure 4A). Given the close association between viral load and disease severity, we further analyzed the correlation between cytokine changes and viral load in male and female patients using Spearman correlation analysis. After BH correction, in male patients, 14 cytokines (CCL4, FGF2, GM-CSF, IL-8, CXCL10, TNF-α, IFN-γ, IL-4, IL-6, IL-1RA, CCL2, CXCL9, CCL11, and G-CSF) showed significant positive correlations with viral load. In female patients, 10 cytokines (CCL4, FGF2, GM-CSF, CXCL10, TNF-α, IFN-γ, IL-6, IL-1RA, CCL2, and CXCL9) displayed significant positive correlations with viral load (Figure 4B–O). We further formally compared the corresponding Spearman correlation coefficients between male and female patients. After BH correction, none of the cytokine–viral load correlation coefficients differed significantly between the two sexes (all BH-adjusted *p* > 0.05) (Table S6).

To directly assess whether the association between viral load and normalized cytokine ratios differed by sex, sex × viral load interactions were tested for all 19 cytokines. After BH correction, none of the 19 cytokines showed a significant sex × viral load interaction (all BH-adjusted *p* > 0.05) (Table S7). Spearman correlation analyses of cytokine changes with other laboratory indicators also identified significant within-sex associations with AST, ALT, LDH, CK, and platelet count after BH correction, whereas no significant correlations with white blood cell count remained after correction (Figures S5–S9, Table S8). Formal between-sex comparisons of these correlation coefficients showed no significant differences for any of the laboratory indicators after BH correction (all BH-adjusted *p* > 0.05) (Table S6).

### 3.6. Sex-Specific Differences and Discriminatory Performance of Cytokines

**Figure 5:**
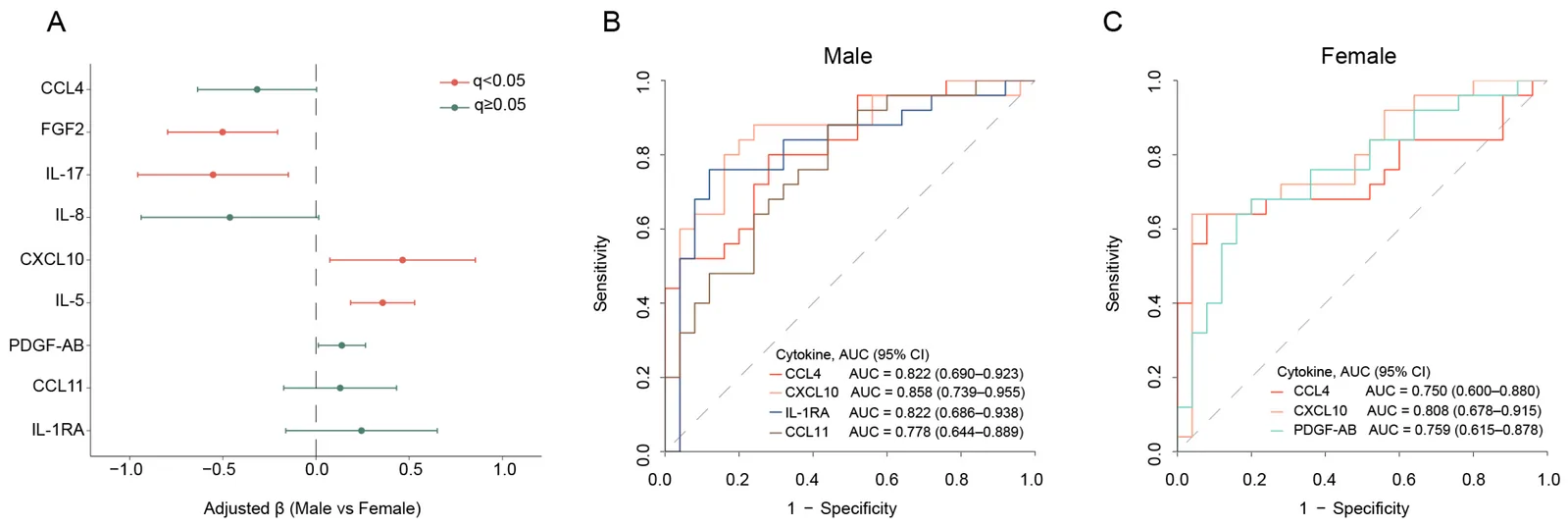
Sex-associated cytokine differences and ROC analyses for discrimination between concurrent mild and severe SFTS. (**A**) Adjusted β coefficients with 95% CIs for sex-associated cytokine differences. Female sex was the reference; positive and negative β values indicate higher cytokine response magnitude in males and females, respectively. Colors indicate significance after Benjamini–Hochberg correction (*q* < 0.05). (**B**,**C**) Sex-stratified ROC curves for discrimination between concurrent mild and severe SFTS in male and female patients, respectively. For graphical clarity, ROC curves with AUC ≥ 0.75 are displayed.

Considering the observed sex-related differences in normalized cytokine ratios, we selected nine cytokines that exhibited significant sex differences in at least one of the BH-adjusted overall or disease-severity-stratified analyses (Figure 3A,B) for exploratory multivariable linear regression. After adjusting for age, interval from symptom onset to sample collection, viral load, disease severity, ALT, LDH, CK, platelet count, and white blood cell count, four of the nine cytokines remained significantly associated with sex after BH correction. Among these, FGF2 [β = −0.501, 95% CI (−0.796, −0.206), BH-adjusted *p* = 0.005] and IL-17 [β = −0.552, 95% CI (−0.956, −0.148), BH-adjusted *p* = 0.024] showed higher normalized cytokine ratios in females, whereas IL-5 [β = 0.357, 95% CI (0.185, 0.529), BH-adjusted *p* < 0.001] and CXCL10 [β = 0.465, 95% CI (0.074, 0.855), BH-adjusted *p* = 0.045] showed higher normalized cytokine ratios in males (Figure 5A, Table S9). Sensitivity analyses excluding the potentially influential observation and using a parsimonious model generally supported the robustness of the principal adjusted associations (Table S10).

To further evaluate the ability of cytokines to discriminate between concurrent mild and severe SFTS, exploratory ROC analyses were performed separately in male and female patients using the complete sex-specific datasets. The nine candidate cytokines identified from the preceding analyses were evaluated individually. In male patients, CXCL10 showed an AUC of 0.858 (95% CI: 0.739–0.955), while CCL4 and IL-1RA each showed an AUC of 0.822 (95% CI: 0.690–0.923 and 0.686–0.938, respectively), and CCL11 showed an AUC of 0.778 (95% CI: 0.644–0.889) (Figure 5B, Table S11). In female patients, CXCL10 showed an AUC of 0.808 (95% CI: 0.678–0.915), PDGF-AB showed an AUC of 0.759 (95% CI: 0.615–0.878), and CCL4 showed an AUC of 0.750 (95% CI: 0.600–0.880) (Figure 5C, Table S11).

## 4. Discussion

This study analyzed sex-specific cytokine response features in patients with SFTS, focusing on cytokine changes in male and female patients across different disease severities and their relationships with viral load and laboratory parameters. Our results indicate that, although the absolute levels of viral load and most clinical parameters showed limited differences between sexes, significant sex-specific differences were observed in the magnitude of cytokine changes relative to sex-matched healthy reference levels for several cytokines.

Cytokines are key signaling molecules that regulate inflammation and antiviral responses. Dysregulated cytokine release can trigger a cytokine storm, resulting in systemic inflammation and tissue damage, a mechanism implicated in the pathogenesis of many severe viral infections, including SFTSV [11,23,24]. In this study, among the 19 cytokines measured, the majority were significantly elevated in SFTS patients, showing a general trend of higher levels in severe versus mild cases and in cases versus healthy controls. PCA revealed distinct clustering of severe patients, mild patients, and controls based on overall cytokine levels, indicating differences in overall cytokine profiles across disease-severity groups and healthy controls. These findings are consistent with previous studies on immune and inflammatory responses in SFTS [25,26].

Notably, direct comparison of absolute cytokine concentrations between male and female patients may not fully capture disease-related immune differences. Some cytokines exhibit significant baseline sex differences in healthy individuals, which can mask or exaggerate sex effects under disease conditions. Therefore, we applied a baseline-normalized analysis, calculating normalized cytokine ratios relative to sex-matched healthy controls using log_2_[(measured value/median of controls) + 1], to better characterize infection-associated changes in cytokine responses. Previous studies have primarily evaluated cytokine alterations and sex-related differences based on absolute cytokine concentrations, whereas the baseline-normalized analysis in the present study provides complementary information by assessing the magnitude of cytokine changes relative to sex-specific healthy reference levels. This analysis revealed that CCL4, IL-17, IL-8, and FGF2 showed higher normalized cytokine ratios in female patients, whereas IL-5 and PDGF-AB showed higher normalized ratios in male patients after BH correction. Further severity-stratified analyses identified additional sex differences, including higher normalized ratios of CXCL10, CCL11, PDGF-AB, IL-5 and IL-1RA in male patients with severe disease. These cytokines are largely involved in immune cell recruitment, antiviral responses, and tissue repair [26,27,28]. PDGF-AB, a growth factor associated with platelet function and vascular repair, may be related to alterations in platelet-associated and vascular responses in SFTS [29]. CXCL10 is an important chemokine mediating the recruitment of inflammatory cells to infection sites and has been strongly linked to disease severity and mortality in SFTS [30,31,32,33]. Among these findings, the higher normalized IL-8 ratio observed in female patients was directionally consistent with previous reports of higher absolute IL-8 concentrations in females, although the two measures represent different aspects of the cytokine response [30]. However, formal sex × disease severity interaction analysis identified a significant interaction only for PDGF-AB after BH correction, suggesting that the sex differences observed within individual severity groups should be interpreted cautiously. Nevertheless, the differences in normalized cytokine ratios observed in the overall and stratified analyses provide complementary information on sex-related cytokine response relative to sex-specific healthy reference levels.

Viral load has long been recognized as a critical determinant of SFTS severity [34,35,36,37]. Although no significant difference in viral load was observed between sexes, a larger number of significant correlations between viral load and cytokine changes were observed in males than in females after BH correction. However, formal comparisons of the corresponding Spearman correlation coefficients showed no significant differences between sexes after BH correction. Similarly, although significant within-sex associations were observed between several cytokines and AST, ALT, LDH, CK, and platelet count, formal between-sex comparisons of these correlations were not significant. Taken together, these findings suggest that viral burden and disease-related laboratory abnormalities are closely associated with cytokine changes during acute SFTS, while the present data provide no clear evidence that these associations are sex-specific.

Sex is an important biological factor influencing both innate and adaptive immune responses [17]. Females typically mount stronger innate and adaptive immune responses, potentially related to X chromosome-linked immune gene dosage and hormonal regulation [16,38,39,40]. Multivariable linear regression analysis further showed that four of the nine candidate cytokines remained significantly associated with sex after adjustment for potential confounders and BH correction. FGF2 and IL-17 showed higher normalized cytokine ratios in females, whereas IL-5 and CXCL10 showed higher normalized ratios in males. These findings further support the presence of sex-related differences in normalized cytokine responses in SFTS, although the underlying biological mechanisms remain to be clarified. In addition, ROC curve analysis is a commonly used method for evaluating the discriminatory performance of biomarkers in distinguishing different clinical outcomes [41]. In the exploratory sex-stratified ROC analyses, several cytokines showed relatively high AUCs for distinguishing concurrent mild and severe disease within each sex. These findings should be considered exploratory and require validation in larger independent cohorts. Previous studies have also reported associations between cytokine and chemokine responses, particularly CXCL10/IP-10 and IL-6, and SFTS disease severity [25,30,42].

This study has several limitations. First, it was a single-center case–control study with a relatively small sample size, which may limit the generalizability of the findings. The balanced sampling design was intended for group comparisons and does not reflect the natural distribution of sex and disease severity in the source population; it was not designed to estimate sex-specific risks of severe disease or population-level biomarker performance. Although some sex-related differences remained after multivariable adjustment, these findings, including the exploratory ROC results, require validation in larger independent multicenter studies. Second, the analysis was based on a single acute-phase serum sample per patient and therefore did not capture dynamic changes in cytokine levels and viral load throughout the disease course, thus limiting our ability to fully characterize the temporal features of sex-specific immune responses. In addition, treatments administered before sample collection may have influenced cytokine levels, although complete treatment information was not consistently available. Third, the study focused on serum cytokine levels without investigating immune cell subsets, tissue-specific immune responses, or molecular mechanisms, and unmeasured demographic, lifestyle, medication-related, and hormonal factors may also have contributed to the observed differences, limiting causal interpretation of the observed sex-related differences.

Despite these limitations, this study provides preliminary clues suggesting that, when evaluating SFTS-related immune responses, sex should be considered alongside the magnitude of cytokine changes. These findings may provide a reference for further understanding sex-related immune responses in SFTS.

## 5. Conclusions

Taken together, this study highlights sex-related differences in cytokine characteristics during acute SFTS, particularly in the magnitude of cytokine changes and their associations with viral load and laboratory indicators. Although cytokine changes were associated with viral load and laboratory indicators within both sexes, these associations did not differ significantly between males and females. These findings suggest that sex and cytokine response magnitude should be considered when characterizing SFTS-related cytokine responses and their associations with disease severity. CXCL10 and CCL4 showed relatively high AUCs for distinguishing concurrent mild and severe disease in both sexes. Further multicenter and longitudinal studies are needed to validate these findings and clarify the biological basis of sex-related differences in cytokine responses in SFTS.

## Data Availability

The data presented in this study are available from the corresponding author upon reasonable request.

## Supplementary Materials

The following supporting information can be downloaded at: https://www.mdpi.com/article/10.3390/microorganisms14102242/s1, Table S1: Demographic and clinical characteristics of all SFTS participants; Table S2: Age distribution of patients with SFTS and healthy controls stratified by sex; Table S3: Detailed statistical results for sex differences in log_2_-transformed normalized cytokine ratios in the overall patient population; Table S4: Sex differences in log_2_-transformed normalized cytokine ratios stratified by disease severity; Table S5: Sex × disease severity interaction analyses for normalized cytokine changes in patients with SFTS; Table S6: Comparison of Spearman correlation coefficients between male and female patients; Table S7: Sex × viral load interaction analyses for normalized cytokine changes in patients with SFTS; Table S8: Sex-stratified Spearman correlations between normalized cytokine ratios and laboratory indicators in patients with SFTS; Table S9: Adjusted associations between sex and normalized cytokine ratios in multivariable linear regression analyses; Table S10: Sensitivity analyses of the adjusted associations between sex and normalized cytokine ratios; Table S11: Exploratory sex-stratified ROC analysis of candidate cytokines for discrimination between concurrent mild and severe SFTS; Figure S1: Flowchart of patient screening and selection for the final analytical cohort; Figure S2: Comparison of cytokine levels among control, mild, and severe groups; Figure S3: Sex-specific differences in normalized cytokine ratios relative to healthy reference levels; Figure S4: Sex-specific differences in normalized cytokine ratios according to disease severity; Figure S5: Associations between AST levels and normalized cytokine ratios; Figure S6: Associations between ALT levels and normalized cytokine ratios; Figure S7: Associations between LDH levels and normalized cytokine ratios; Figure S8: Associations between CK levels and normalized cytokine ratios; Figure S9: Associations between platelet counts and normalized cytokine ratios.

## Abbreviations

The following abbreviations are used in this manuscript:

SFTS	Severe Fever with Thrombocytopenia Syndrome
SFTSV	Severe Fever with Thrombocytopenia Syndrome Virus
RT-qPCR	Reverse Transcription Quantitative Polymerase Chain Reaction
PCA	Principal Component Analysis
ROC	Receiver Operating Characteristic
AUC	Area Under the Curve
SD	Standard Deviation
