# Supplementary material for: Sex-Specific Cytokine Profiles Associated with Viral Load and Disease Severity in Patients with Severe Fever with Thrombocytopenia Syndrome
Source: Microorganisms. 2026 Oct 3;14(10):2242. doi: 10.3390/microorganisms14102242 (PMC13707548; doi:10.3390/microorganisms14102242)
Supplement: Supplementary file 1 [file microorganisms-14-02242-s001.zip › Supplemental figures S1-S9.pdf]

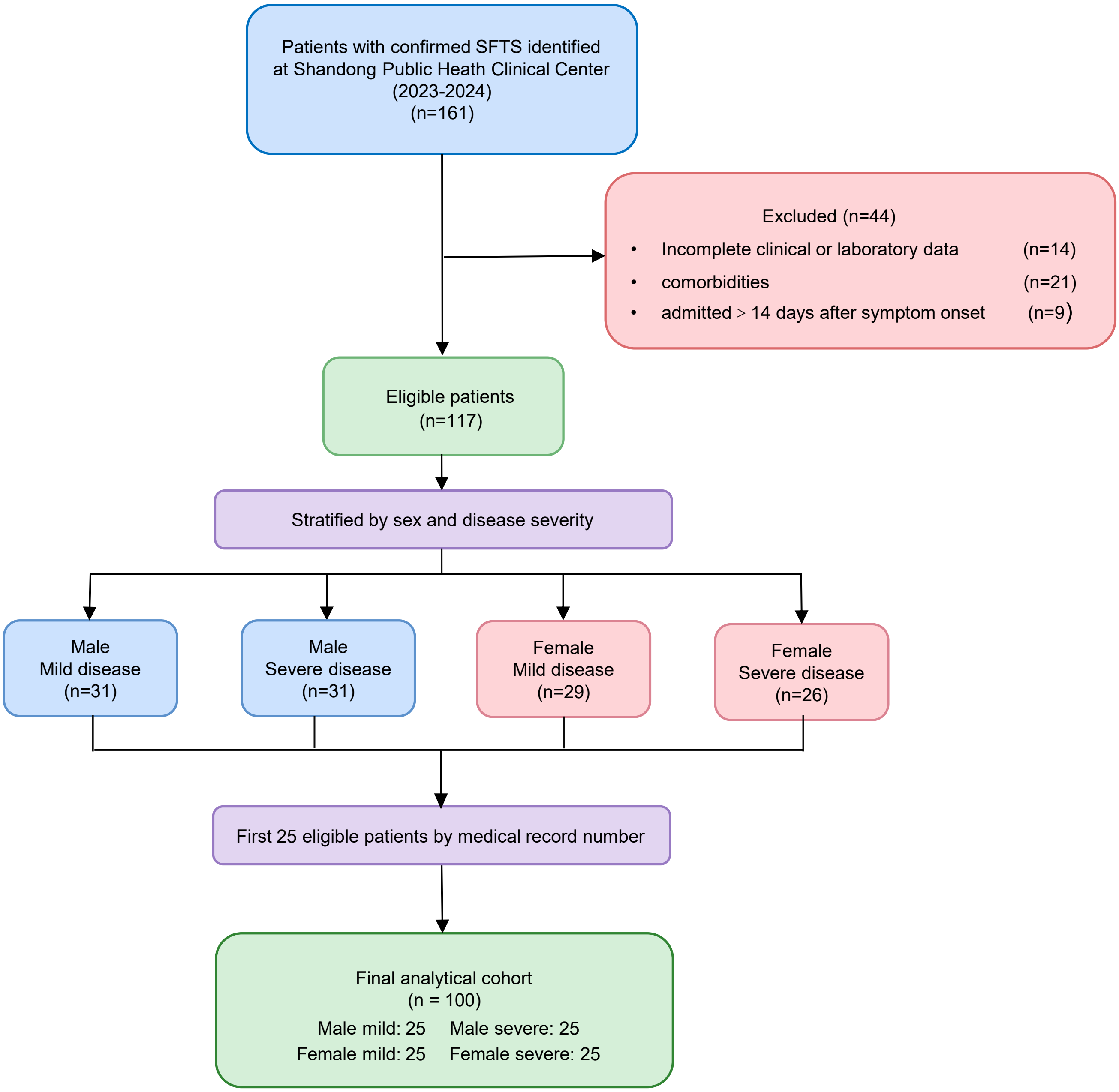

Supplementary Figure S1. Flowchart of patient screening and selection for the final analytical cohort.

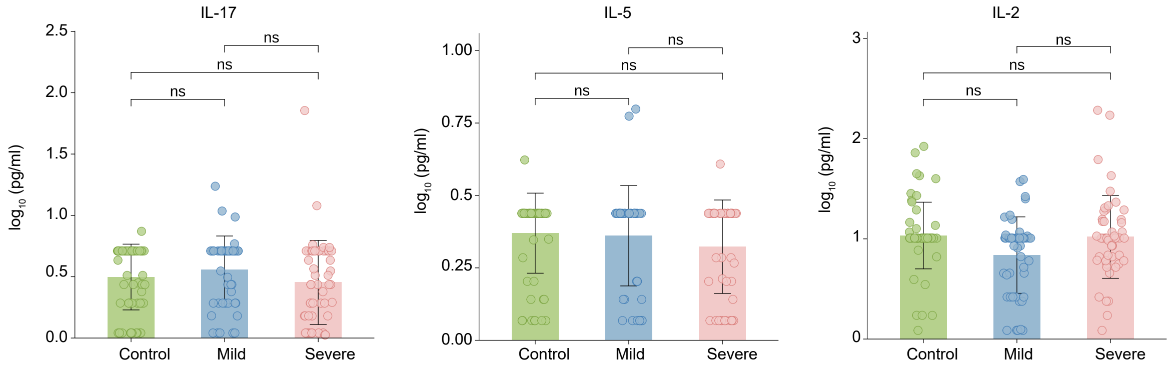

Supplementary Figure S2. Comparison of cytokine levels among control, mild, and severe groups. IL-17, IL-5 , and IL-2 showed no significant differences between mild and severe cases or between mild and/or severe cases and controls. For visualization purposes, data are presented as mean ± SD, and statistical comparisons among groups were performed using the Mann-Whitney *U* test with Benjamini-Hochberg correction.

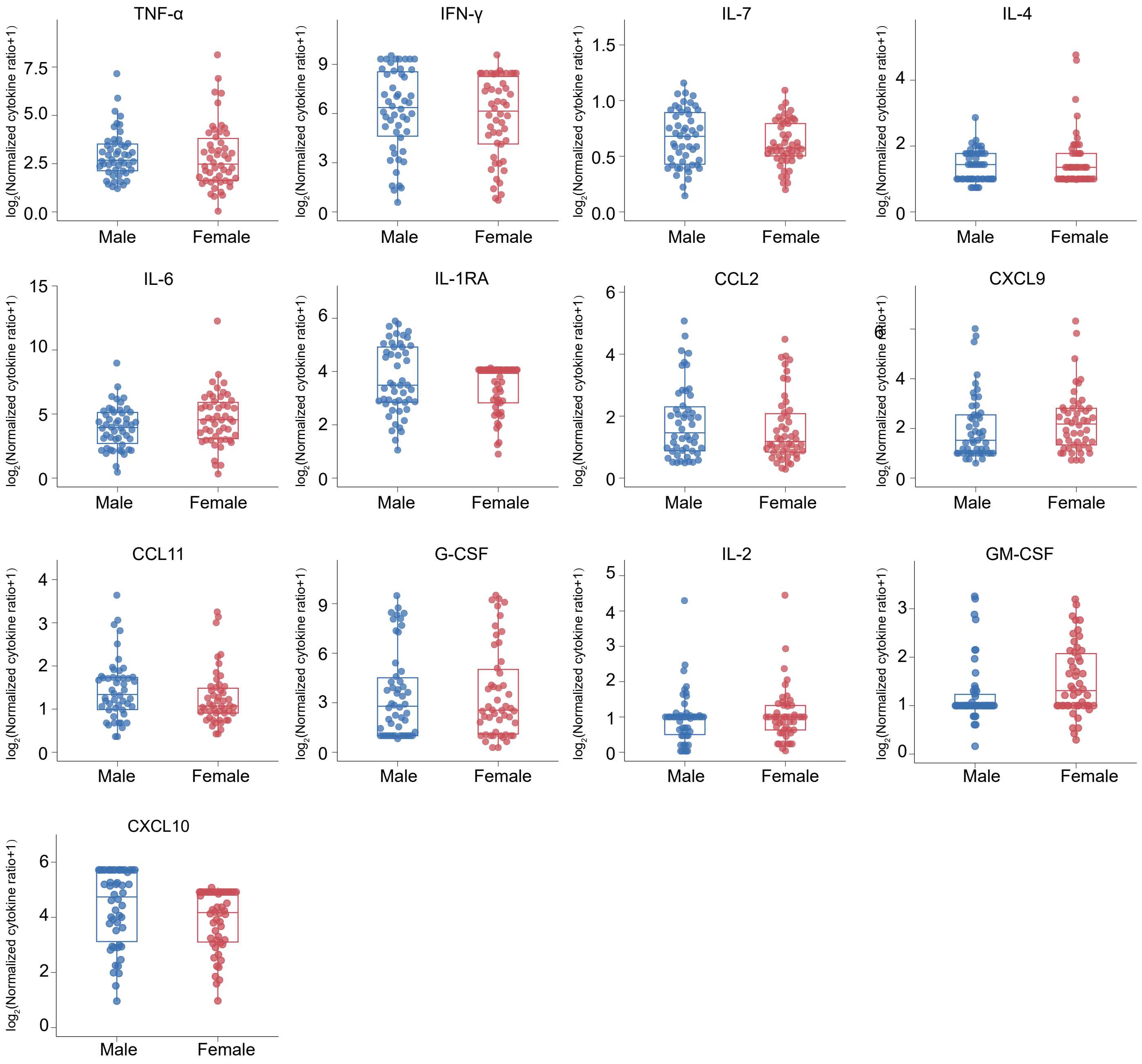

Supplementary Figure S3. Sex-specific differences in normalized cytokine ratios relative to healthy reference levels. Thirteen cytokines showed no significant differences between males and females. Statistical comparisons were performed using the Mann-Whitney *U* test with Benjamini-Hochberg correction.

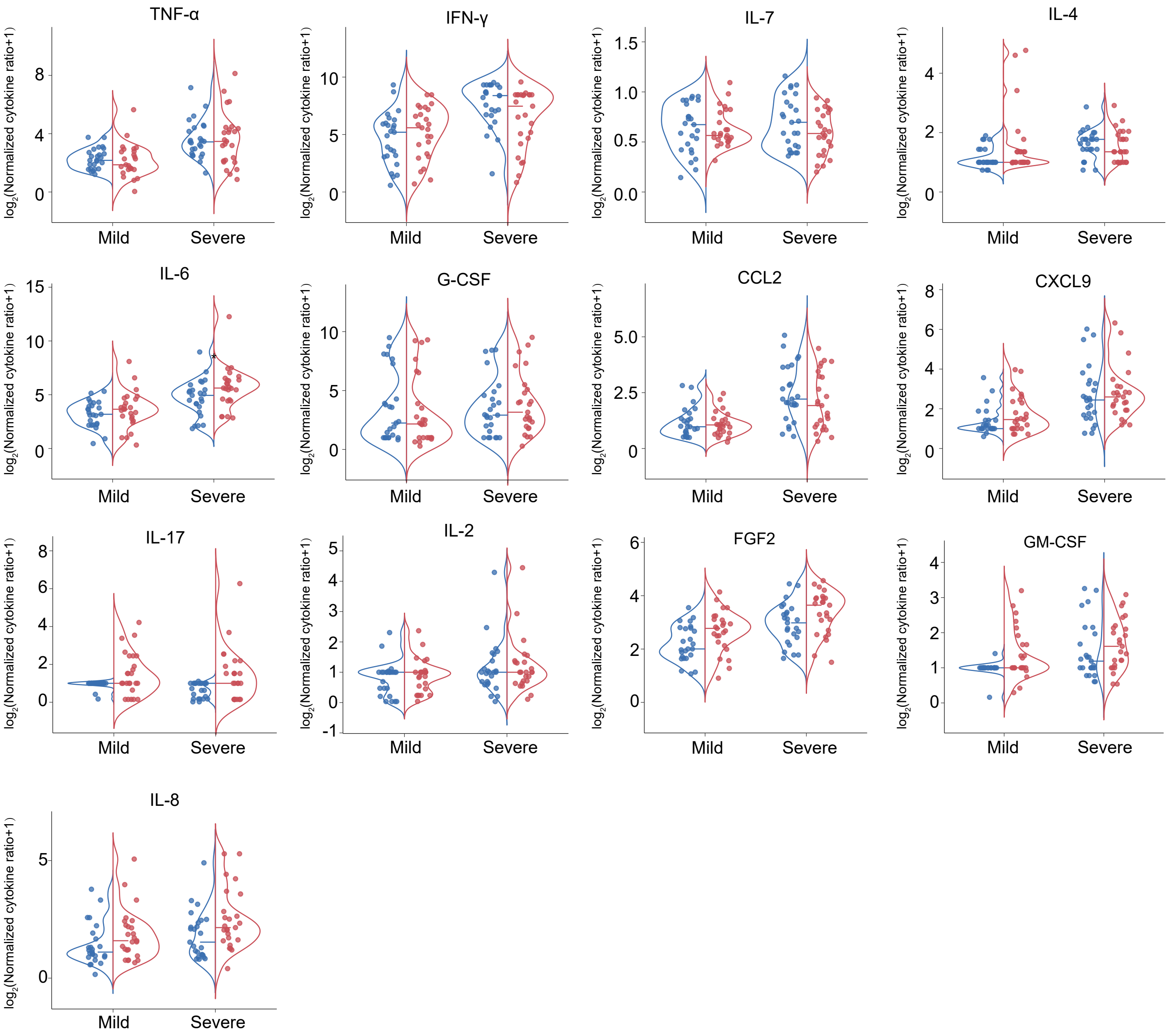

Supplementary Figure S4. Sex-specific differences in normalized cytokine ratios according to disease severity. Normalized cytokine ratios were compared between male and female patients in the mild and severe groups. Statistical comparisons were performed using the Mann-Whitney *U* test with Benjamini-Hochberg correction.

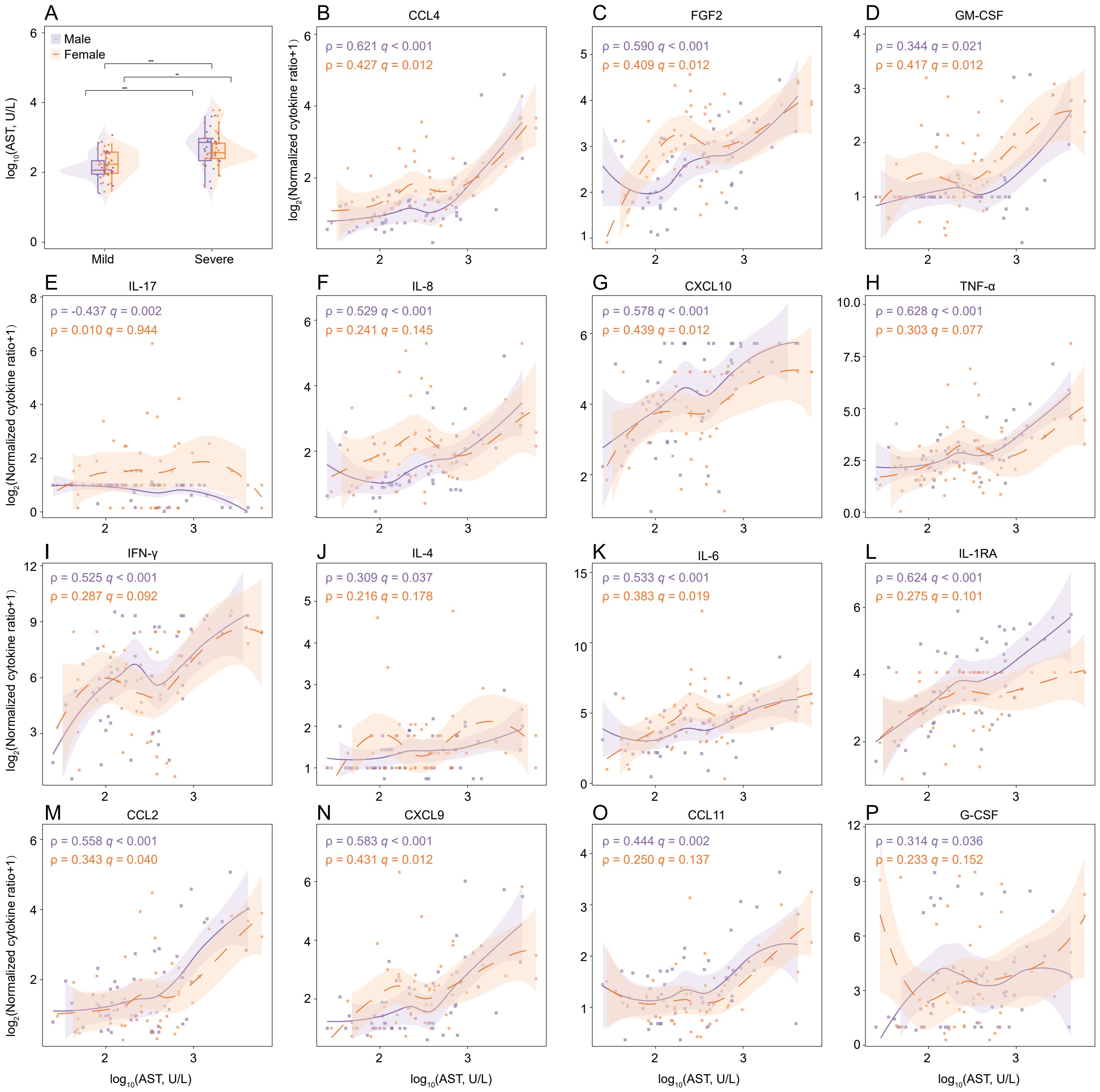

Supplementary Figure S5. Associations between AST levels and normalized cytokine ratios. (A) Comparisons of AST levels between mild and severe cases in male and female patients separately and in the overall cohort. AST levels were log10-transformed. (B-P) Spearman correlation analyses between AST levels and normalized cytokine ratios in male and female patients. *q* values were obtained using the Benjamini-Hochberg correction.

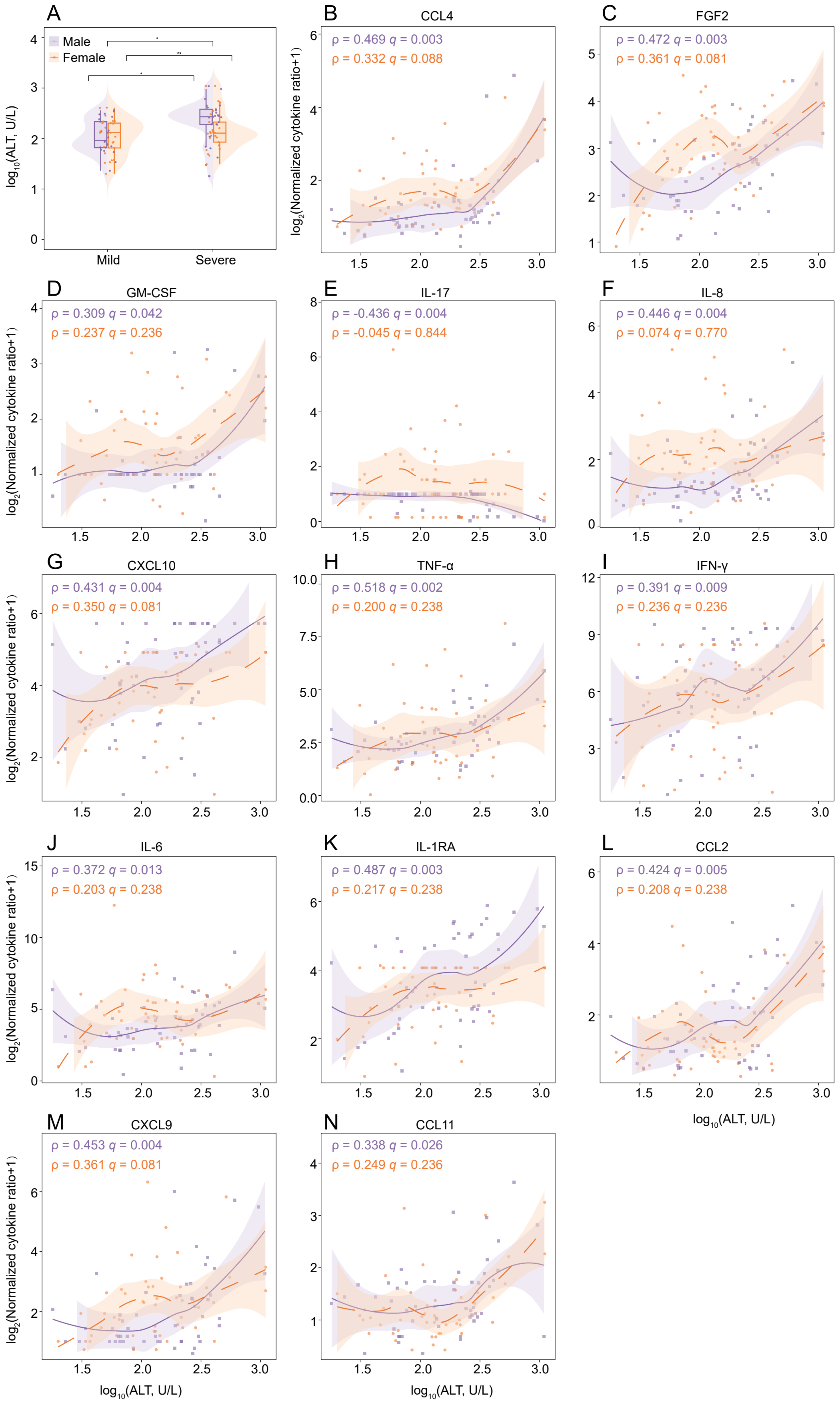

Supplementary Figure S6. Associations between ALT levels and normalized cytokine ratios. (A) Comparisons of ALT levels between mild and severe cases in male and female patients separately and in the overall cohort. ALT levels were log10-transformed. (B-N) Spearman correlation analyses between ALT levels and normalized cytokine ratios in male and female patients. *q* values were obtained using the Benjamini-Hochberg correction.

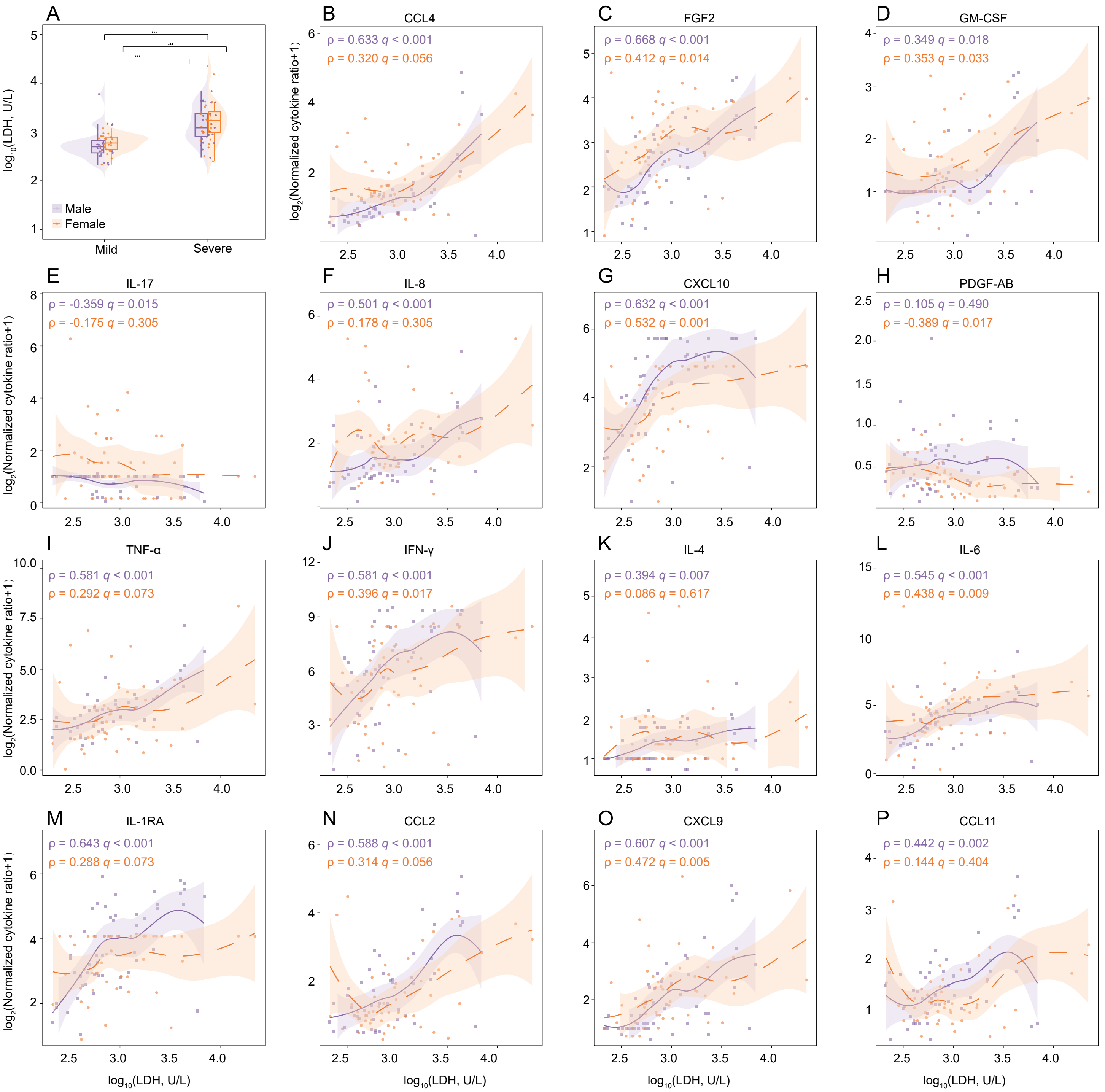

Supplementary Figure S7. Associations between LDH levels and normalized cytokine ratios. (A) Comparisons of LDH leve ls between mild and severe cases in male and female patients separately and in the overall cohort. LDH levels were log10-transformed. (B-P) Spearman correlation analyses between LDH levels and normalized cytokine ratios in male and female patients. *q* values were obtained using the Benjamini-Hochberg correction.

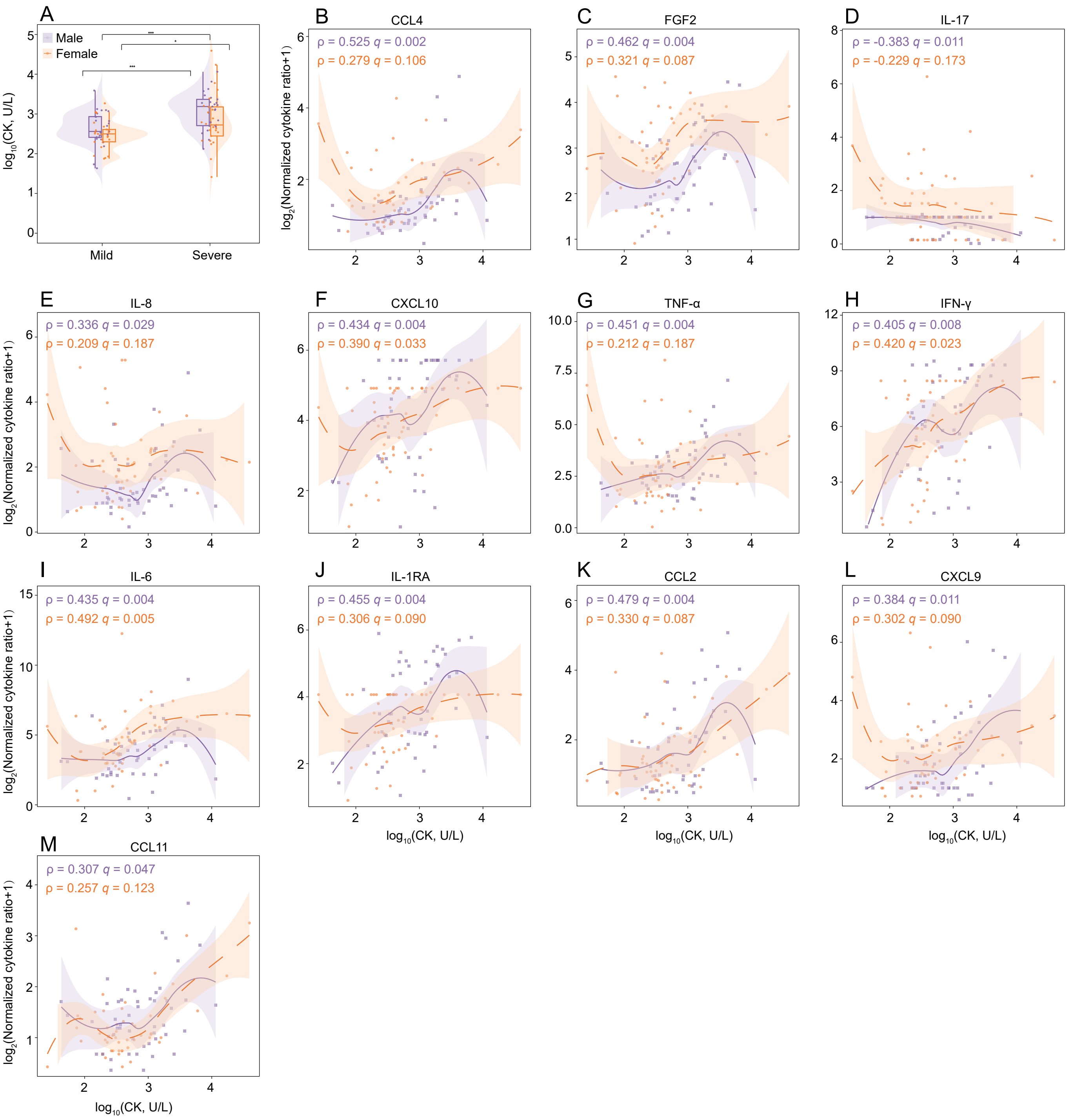

Supplementary Figure S8. Associations between CK levels and normalized cytokine ratios. (A) Comparisons of CK levels betwee n mild and severe cases in male and female patients separately and in the overall cohort. CK levels were log10-transformed. (B-M) Spearman correlation analyses between CK levels and normalized cytokine ratios in male and female patients. *q* values were obtained using the Benjamini-Hochberg correction.

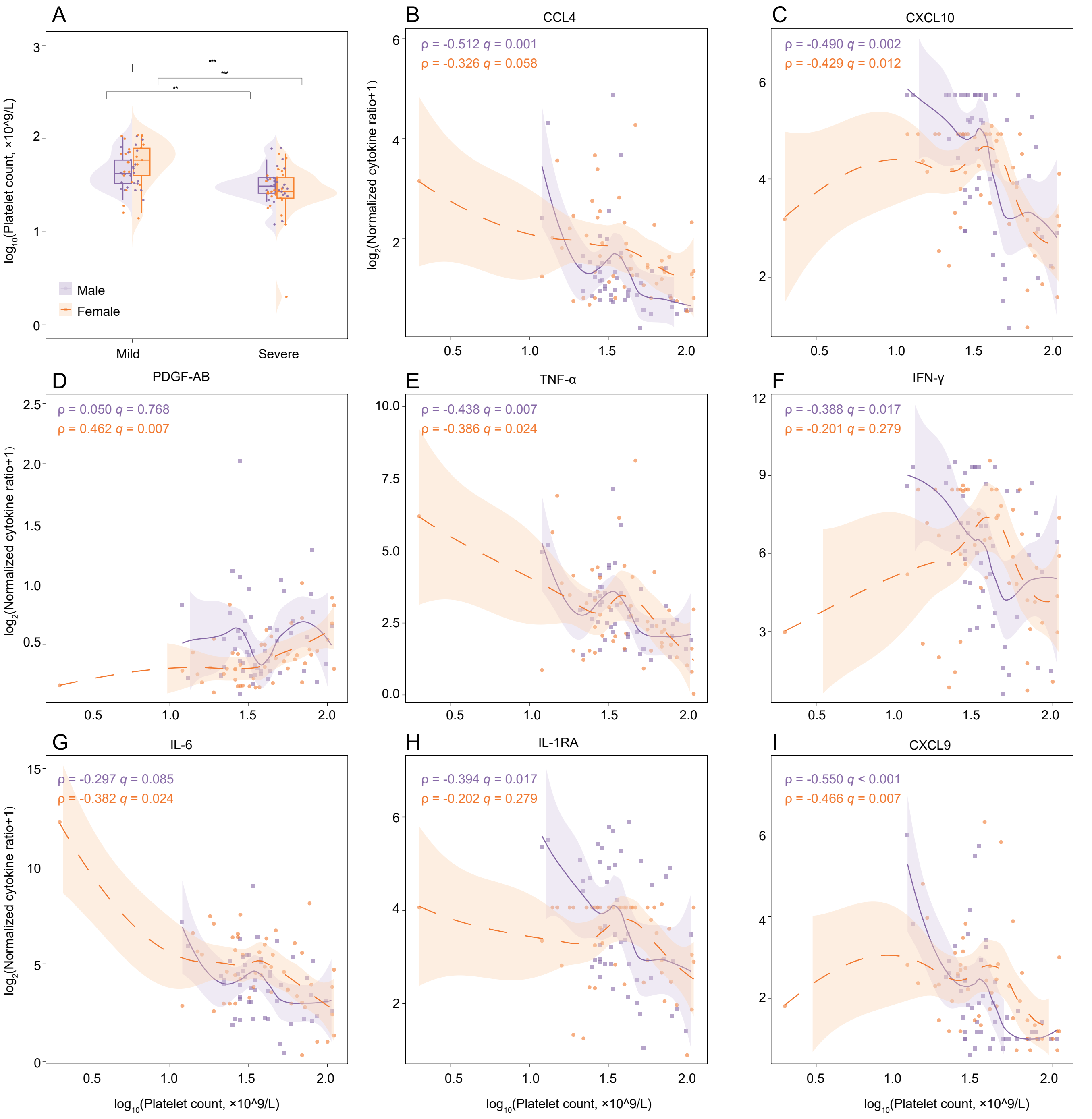

Supplementary Figure S9. Associations between platelet counts and normalized cytokine ratios. (A) Comparisons of platele t counts between mild and severe cases in male and female patients separately and in the overall cohort. Platelet counts wer e log10-transformed. (B-I) Spearman correlation analyses between platelet counts and normalized cytokine ratios in male and female patients. *q* values were obtained using the Benjamini-Hochberg correction.
