# Supplementary material for: Sex-Specific Cytokine Profiles Associated with Viral Load and Disease Severity in Patients with Severe Fever with Thrombocytopenia Syndrome
Source: Microorganisms. 2026 Oct 3;14(10):2242. doi: 10.3390/microorganisms14102242 (PMC13707548; doi:10.3390/microorganisms14102242)
Supplement: Supplementary file 1 [file microorganisms-14-02242-s001.zip › Supplemental Table S1.pdf]

Supplemental Table S1 Demographic and clinical characteristics of all SFTS participants.

	Mild		Severe		*P* values[a]	*P* values[b]	*P* values[c]
	Male (n=25)	Female (n=25)	Male (n=25)	Female (n=25)			
Age (years) median (IQR)	67 (56-75)	60 (53-69)	70 (68-74)	66 (58-72)	0.174	0.016~~<0.05~~	0.008~~<0.01~~
Interval from symptom onset to admission (days) median (IQR)	6 (5-7)	6 (5-7)	5 (4-7)	7 (5-7)	0.883	0.250	0.433
History of tick bite n (%)	12 (48)	7 (28)	7 (28)	8 (32)	0.145	0.758	0.398
Occupation as farmer n (%)	15 (60)	14 (56)	15 (60)	9 (36)	0.774	0.089	0.161
**Clinical manifestations n (%)**							
Fever	23 (92)	25 (100)	24 (96)	23 (92)	0.49	1.000	1.000
Headache	5 (20)	3 (12)	6 (24)	1 (4)	0.702	0.098	0.091
Dizziness	2 (8)	2 (8)	6 (24)	3 (12)	1.000	0.463	0.372
Shiver	1 (4)	3 (12)	5 (20)	1 (4)	0.609	0.189	0.741
Fatigue	4 (16)	3 (12)	8 (32)	3 (12)	1.000	0.171	0.118
Myalgia	11 (44)	6 (24)	8 (32)	6 (24)	0.136	0.529	0.130
Malaise	15 (60)	17 (68)	15 (60)	17 (68)	0.556	0.556	0.405
Conjunctival hemorrhage	0 (0)	0 (0)	2 (8)	0 (0)	1.000	0.49	0.495
Skin ecchymosis	0 (0)	0 (0)	6 (24)	5 (20)	1.000	0.733	0.749
Gingival bleeding	0 (0)	0 (0)	2 (8)	4 (16)	1.000	0.667	0.678
**Gastrointestinal manifestations n (%)**							
Poor appetite	19 (76)	22 (88)	18 (72)	20 (80)	0.463	0.508	0.220
Nausea	5 (20)	8 (32)	7 (28)	9 (36)	0.333	0.544	0.271
Vomiting	4 (16)	9 (36)	5 (20)	6 (24)	0.196	0.733	0.160
Hematemesis	0 (0)	0 (0)	7 (28)	2 (8)	1.000	0.138	0.160
Abdominal Pain	1 (4)	0 (0)	3 (12)	2 (8)	1.000	1.000	0.678
Abdominal distension	1 (4)	3 (12)	3 (12)	2 (8)	0.609	1.000	1.000
Diarrhea	5 (20)	4 (16)	8 (32)	10 (40)	1.000	0.556	0.822
Hematochezia	0 (0)	0 (0)	5 (20)	7 (28)	1.000	0.508	0.538
**Neurological manifestations n (%)**							
Coma	0 (0)	0 (0)	8 (32)	9 (36)	1.000	0.765	0.790
Shock	0 (0)	0 (0)	4 (16)	5 (20)	1.000	1.000	1.000
Somnolence	0 (0)	0 (0)	9 (36)	10 (40)	1.000	0.771	0.799
Confusion	0 (0)	0 (0)	11 (44)	8 (32)	1.000	0.382	0.444
Convulsions	0 (0)	0 (0)	4 (16)	1 (4)	1.000	0.349	0.362
**Laboratory findings median (IQR)**							
PLT count, $\times 10^9$/L	41 (32-58)	58 (39-78)	30 (25-37)	26 (22-37)	0.111	0.290	0.901
WBC count, $\times 10^9$/L	1.61 (1.37-1.92)	1.91 (1.41-2.59)	2.18 (1.43-2.38)	1.63 (1.12-2.6)	0.225	0.712	0.600
AST level, U/L	115 (88-215)	170 (93-380)	722 (216-942)	365 (249-681)	0.286	0.509	0.833
ALT level, U/L	90 (65-215)	130 (64-201)	269 (188-379)	127 (84-211)	1.000	0.091	0.220
LDH level, U/L	496 (372-666)	593 (437-786)	1214 (800-2355)	1714 (969-2600)	0.177	0.749	0.556
CK level, U/L	362 (257-856)	314 (198-406)	1536 (507-2328)	527 (278-1504)	0.194	0.060	0.020

Viral load ((log$_{10}$ copies/ul)	2.55(1.98-3.67)	2.98 (2.05-3.35)	4.24 (3.67-5.24)	4.73 (3.42-5.83)	0.984	0.497	0.681
**Outcomes n (%)**							
Survival	25	25	15	16			
Death	0	0	10	9			

Notes: *P* values[a]: Comparison between male and female patients with mild SFTS. *P* values[b]: Comparison between male and female patients with severe SFTS. *P* values[c]: Comparison between male and female patients with SFTS. Data are presented as median (interquartile range) or percentages. Continuous variables were compared using the Mann-Whitney *U* test, and categorical variables were compared using the χ^2 test or Fisher's exact test.

Abbreviations: PLT, platelet; WBC, white blood cell; AST, aspartate aminotransferase; ALT, alanine aminotransferase; LDH, lactate dehydrogenase; CK, creatine kinase.
