# Supplementary material for: Sex-Specific Cytokine Profiles Associated with Viral Load and Disease Severity in Patients with Severe Fever with Thrombocytopenia Syndrome
Source: Microorganisms. 2026 Oct 3;14(10):2242. doi: 10.3390/microorganisms14102242 (PMC13707548; doi:10.3390/microorganisms14102242)
Supplement: Supplementary file 1 [file microorganisms-14-02242-s001.zip › Supplemental Table S10.pdf]

Supplementary Table S10. Sensitivity analyses of the adjusted associations between sex and normalized cytokine ratios.

Cytokine	Excluding potentially influential observation (N = 99)		Parsimonious model (N = 100)	
	β (95% CI)	BH-adjusted *P*	β (95% CI)	BH-adjusted *P*
CCL4	−0.282 (−0.606, 0.042)	0.1128	−0.330 (−0.658, −0.003)	0.0618
FGF2	−0.485 (−0.783, −0.188)	0.0076	−0.446 (−0.750, −0.141)	0.0137
IL-17	−0.481 (−0.886, −0.076)	0.0497	−0.649 (−1.033, −0.266)	0.0050
IL-8	−0.384 (−0.822, 0.055)	0.1128	−0.524 (−0.996, −0.052)	0.0450
IL-5	0.346 (0.174, 0.518)	0.0012	0.340 (0.183, 0.497)	0.0004
PDGF-AB	0.143 (0.016, 0.270)	0.0497	0.168 (0.046, 0.291)	0.0139
CXCL10	0.451 (0.060, 0.842)	0.0497	0.524 (0.151, 0.897)	0.0139
CCL11	0.130 (−0.179, 0.438)	0.4062	0.229 (−0.033, 0.491)	0.0859
IL-1RA	0.278 (−0.129, 0.685)	0.2001	0.361 (−0.049, 0.771)	0.0859

Note: Female sex was used as the reference group; negative β values indicate higher normalized cytokine ratios in females, whereas positive β values indicate higher ratios in males. *P* values were adjusted using the Benjamini-Hochberg (BH) procedure.
