# Supplementary material for: Sex-Specific Cytokine Profiles Associated with Viral Load and Disease Severity in Patients with Severe Fever with Thrombocytopenia Syndrome
Source: Microorganisms. 2026 Oct 3;14(10):2242. doi: 10.3390/microorganisms14102242 (PMC13707548; doi:10.3390/microorganisms14102242)
Supplement: Supplementary file 1 [file microorganisms-14-02242-s001.zip › Supplemental Table S11.pdf]

Supplementary Table S11. Exploratory sex-stratified ROC analysis of candidate cytokines for discrimination between concurrent mild and severe SFTS.

Sex	Cytokine	N	AUC (95% CI)	Cutoff (95% CI)	Sensitivity (95% CI)	Specificity (95% CI)
Male	CCL4	50	0.822 (0.690–0.923)	1.015 (0.807–1.519)	0.800 (0.440–1.000)	0.720 (0.480–1.000)
Male	FGF2	50	0.748 (0.608–0.867)	2.401 (2.077–3.236)	0.760 (0.400–0.960)	0.640 (0.480–1.000)
Male	IL-17	50	0.683 (0.562–0.805)	0.859 (0.359–0.859)	0.480 (0.280–0.680)	0.920 (0.800–1.000)
Male	IL-8	50	0.617 (0.451–0.765)	1.351 (0.715–2.005)	0.560 (0.400–1.000)	0.720 (0.160–0.920)
Male	CXCL10	50	0.858 (0.739–0.955)	4.854 (4.217–5.448)	0.800 (0.640–1.000)	0.840 (0.680–1.000)
Male	IL-5	50	0.522 (0.423–0.620)	0.366 (0.366–1.231)	0.960 (0.040–1.000)	0.120 (0.040–1.000)
Male	PDGF-AB	50	0.676 (0.518–0.814)	0.390 (0.239–0.953)	0.720 (0.240–1.000)	0.560 (0.320–1.000)
Male	IL-1RA	50	0.822 (0.686–0.938)	3.611 (3.107–4.524)	0.760 (0.560–0.920)	0.880 (0.720–1.000)
Male	CCL11	50	0.778 (0.644–0.889)	1.110 (0.932–1.739)	0.880 (0.480–1.000)	0.560 (0.400–0.960)
Female	CCL4	50	0.750 (0.600–0.880)	1.853 (1.542–2.142)	0.640 (0.440–0.840)	0.920 (0.800–1.000)
Female	FGF2	50	0.735 (0.584–0.864)	3.237 (2.985–3.653)	0.640 (0.400–0.880)	0.800 (0.640–1.000)
Female	IL-17	50	0.545 (0.393–0.700)	0.394 (0.394–2.962)	0.320 (0.240–1.000)	0.800 (0.120–0.920)
Female	IL-8	50	0.675 (0.515–0.816)	1.961 (1.195–2.599)	0.640 (0.360–1.000)	0.640 (0.280–0.960)
Female	CXCL10	50	0.808 (0.678–0.915)	4.882 (3.124–4.882)	0.640 (0.480–0.920)	0.960 (0.640–1.000)
Female	IL-5	50	0.620 (0.478–0.765)	0.808 (0.280–1.470)	0.680 (0.480–1.000)	0.560 (0.080–0.800)
Female	PDGF-AB	50	0.759 (0.615–0.878)	0.309 (0.275–0.438)	0.680 (0.480–0.920)	0.800 (0.560–0.960)
Female	IL-1RA	50	0.714 (0.566–0.846)	3.931 (3.092–3.931)	0.720 (0.600–0.960)	0.720 (0.440–0.880)
Female	CCL11	50	0.535 (0.358–0.694)	0.709 (0.673–1.242)	0.240 (0.120–0.760)	1.000 (0.520–1.000)

Note: AUC, cutoff, sensitivity, and specificity are presented with 95% confidence intervals estimated from 2,000 stratified bootstrap resamples. Cutoffs were determined using the Youden index and should be considered exploratory rather than clinically validated thresholds.
