# Supplementary material for: Sex-Specific Cytokine Profiles Associated with Viral Load and Disease Severity in Patients with Severe Fever with Thrombocytopenia Syndrome
Source: Microorganisms. 2026 Oct 3;14(10):2242. doi: 10.3390/microorganisms14102242 (PMC13707548; doi:10.3390/microorganisms14102242)
Supplement: Supplementary file 1 [file microorganisms-14-02242-s001.zip › Supplemental Table S2.pdf]

Supplementary Table S2. Age distribution of patients with SFTS and healthy controls stratified by sex

Sex	SFTS patients, n	Patient age, median (IQR)	Healthy controls, n	Control age, median (IQR)	*P*
Male	50	69 (65-75)	30	66 (65-71)	0.170
Female	50	64 (54-70)	30	61 (56-67)	0.578

Data are presented as median (interquartile range). Age distributions between patients with SFTS and healthy controls within each sex were compared using the Mann–Whitney *U* test.
