# Supplementary material for: Sex-Specific Cytokine Profiles Associated with Viral Load and Disease Severity in Patients with Severe Fever with Thrombocytopenia Syndrome
Source: Microorganisms. 2026 Oct 3;14(10):2242. doi: 10.3390/microorganisms14102242 (PMC13707548; doi:10.3390/microorganisms14102242)
Supplement: Supplementary file 1 [file microorganisms-14-02242-s001.zip › Supplemental Table S3.pdf]

Supplementary Table S3. Detailed statistical results for sex differences in log2-transformed normalized cytokine ratios in the overall patient population.

Cytokine	Cliff delta	95% CI	Raw *P*	BH-adjusted *P*
CCL11	0.201	(-0.032, 0.413)	0.084	0.160
CCL2	0.11	(-0.119, 0.329)	0.343	0.461
CCL4	-0.32	(-0.517, -0.091)	0.006	0.043
CXCL10	0.266	(0.026, 0.476)	0.022	0.052
CXCL9	-0.168	(-0.381, 0.062)	0.148	0.256
FGF2	-0.294	(-0.493, -0.067)	0.011	0.043
G-CSF	-0.002	(-0.224, 0.219)	0.986	0.986
GM-CSF	-0.258	(-0.461, -0.030)	0.021	0.052
IFN-r	0.106	(-0.120, 0.321)	0.364	0.461
IL-17	-0.294	(-0.471, -0.093)	0.008	0.043
IL-1RA	0.147	(-0.095, 0.373)	0.203	0.322
IL-2	-0.063	(-0.280, 0.160)	0.585	0.653
IL-4	-0.045	(-0.253, 0.168)	0.693	0.732
IL-5	0.429	(0.248, 0.581)	<0.001	<0.001
IL-6	-0.214	(-0.421, 0.015)	0.066	0.140
IL-7	0.118	(-0.114, 0.339)	0.309	0.452
IL-8	-0.295	(-0.493, -0.069)	0.011	0.043
PDGF-AB	0.284	(0.056, 0.484)	0.015	0.046
TNF-a	0.096	(-0.131, 0.314)	0.410	0.487

Note: Comparisons between male and female patients were performed using the Mann-Whitney *U* test. P values were adjusted across the 19 cytokines using the Benjamini-Hochberg method. Effect sizes are presented as Cliff delta with 95% confidence intervals. Positive Cliff delta values indicate higher values in males, whereas negative Cliff delta values indicate higher values in females.
