# Supplementary material for: Sex-Specific Cytokine Profiles Associated with Viral Load and Disease Severity in Patients with Severe Fever with Thrombocytopenia Syndrome
Source: Microorganisms. 2026 Oct 3;14(10):2242. doi: 10.3390/microorganisms14102242 (PMC13707548; doi:10.3390/microorganisms14102242)
Supplement: Supplementary file 1 [file microorganisms-14-02242-s001.zip › Supplemental Table S4.pdf]

Supplementary Table S4. Sex differences in log2-transformed normalized cytokine ratios stratified by disease severity.

Analysis	Cytokine	Cliff delta	95% CI	Raw *P*	BH-adjusted *P*
Mild	CCL11	-0.133	(-0.441, 0.203)	0.426	0.657
Mild	CCL2	0.021	(-0.299, 0.336)	0.907	0.907
Mild	CCL4	-0.51	(-0.727, -0.201)	0.002	0.039
Mild	CXCL10	0.056	(-0.267, 0.367)	0.742	0.829
Mild	CXCL9	-0.23	(-0.515, 0.101)	0.162	0.440
Mild	FGF2	-0.347	(-0.606, -0.022)	0.036	0.273
Mild	G-CSF	0.107	(-0.216, 0.409)	0.518	0.657
Mild	GM-CSF	-0.283	(-0.505, -0.026)	0.043	0.273
Mild	IFN-γ	-0.146	(-0.445, 0.183)	0.383	0.657
Mild	IL-17	-0.269	(-0.503, 0.002)	0.067	0.314
Mild	IL-1RA	-0.12	(-0.427, 0.212)	0.472	0.657
Mild	IL-2	-0.077	(-0.373, 0.234)	0.640	0.760
Mild	IL-4	-0.189	(-0.432, 0.080)	0.211	0.500
Mild	IL-5	0.238	(-0.002, 0.453)	0.088	0.314
Mild	IL-6	-0.149	(-0.428, 0.157)	0.372	0.657
Mild	IL-7	0.03	(-0.281, 0.336)	0.861	0.907
Mild	IL-8	-0.274	(-0.547, 0.052)	0.099	0.314
Mild	PDGF-AB	-0.133	(-0.429, 0.189)	0.426	0.657
Mild	TNF-a	0.117	(-0.187, 0.400)	0.485	0.657
Severe	CCL11	0.421	(0.076, 0.676)	0.011	0.042
Severe	CCL2	0.184	(-0.146, 0.477)	0.269	0.365
Severe	CCL4	-0.258	(-0.547, 0.087)	0.121	0.208
Severe	CXCL10	0.626	(0.274, 0.830)	<0.001	<0.001
Severe	CXCL9	-0.082	(-0.383, 0.236)	0.628	0.701
Severe	FGF2	-0.306	(-0.577, 0.026)	0.065	0.124
Severe	G-CSF	-0.091	(-0.383, 0.218)	0.587	0.701
Severe	GM-CSF	-0.187	(-0.484, 0.148)	0.259	0.365
Severe	IFN-γ	0.315	(0.002, 0.572)	0.057	0.124
Severe	IL-17	-0.299	(-0.551, 0.003)	0.065	0.124
Severe	IL-1RA	0.53	(0.160, 0.769)	0.001	0.006
Severe	IL-2	-0.046	(-0.349, 0.265)	0.785	0.785
Severe	IL-4	0.083	(-0.242, 0.392)	0.618	0.701
Severe	IL-5	0.616	(0.358, 0.787)	<0.001	<0.001
Severe	IL-6	-0.341	(-0.603, -0.013)	0.040	0.108
Severe	IL-7	0.222	(-0.107, 0.508)	0.180	0.286
Severe	IL-8	-0.341	(-0.598, -0.02)	0.040	0.108
Severe	PDGF-AB	0.638	(0.358, 0.813)	<0.001	<0.001
Severe	TNF-a	0.062	(-0.256, 0.369)	0.712	0.752

Note: Sex differences were assessed using the Mann-Whitney *U* test. *P* values were adjusted across the 19 cytokines within each severity group using the Benjamini-Hochberg (BH) method. Effect sizes are presented as Cliff delta with 95% CIs; positive and negative Cliff delta values indicate higher values in males and females, respectively.
