# Supplementary material for: Sex-Specific Cytokine Profiles Associated with Viral Load and Disease Severity in Patients with Severe Fever with Thrombocytopenia Syndrome
Source: Microorganisms. 2026 Oct 3;14(10):2242. doi: 10.3390/microorganisms14102242 (PMC13707548; doi:10.3390/microorganisms14102242)
Supplement: Supplementary file 1 [file microorganisms-14-02242-s001.zip › Supplemental Table S5.pdf]

Table S5. Sex × disease severity interaction analyses for normalized cytokine changes in patients with SFTS.

| Cytokine | N | Interaction Estimate | 95% CI | *P* value | BH-adjusted *P* value |
|---|---|---|---|---|---|
| TNF-α | 100 | -0.113 | (-1.171, 0.946) | 0.833 | 0.989 |
| IL-6 | 100 | -0.549 | (-1.901, 0.804) | 0.423 | 0.803 |
| IL-8 | 100 | -0.226 | (-1.089, 0.637) | 0.605 | 0.820 |
| IL-7 | 100 | 0.109 | (-0.077, 0.295) | 0.247 | 0.587 |
| CXCL10 | 100 | 0.553 | (-0.308, 1.415) | 0.205 | 0.558 |
| CCL2 | 100 | 0.287 | (-0.513, 1.087) | 0.479 | 0.820 |
| IFN-γ | 100 | 1.798 | (-0.064, 3.660) | 0.058 | 0.277 |
| IL-1RA | 100 | 0.859 | (0.075, 1.642) | 0.032 | 0.277 |
| PDGF-AB | 100 | 0.409 | (0.186, 0.632) | <0.001 | 0.008 |
| CCL4 | 100 | 0.000 | (-0.635, 0.635) | 1.000 | 1.000 |
| IL-4 | 100 | 0.470 | (-0.073, 1.014) | 0.089 | 0.282 |
| IL-17 | 100 | -0.173 | (-0.923, 0.576) | 0.648 | 0.820 |
| IL-2 | 100 | 0.004 | (-0.591, 0.600) | 0.988 | 1.000 |
| GM-CSF | 100 | 0.220 | (-0.314, 0.754) | 0.416 | 0.803 |
| FGF2 | 100 | 0.043 | (-0.578, 0.664) | 0.891 | 0.996 |
| CXCL9 | 100 | 0.295 | (-0.627, 1.217) | 0.527 | 0.820 |
| IL-5 | 100 | 0.264 | (-0.035, 0.564) | 0.083 | 0.282 |
| G-CSF | 100 | -0.623 | (-2.846, 1.600) | 0.579 | 0.820 |
| CCL11 | 100 | 0.499 | (-0.011, 1.009) | 0.055 | 0.277 |

Note: Interaction estimates represent $(\text{severe} - \text{mild})_{male} - (\text{severe} - \text{mild})_{female}$, with female sex and mild disease as the reference categories. Statistical inference was based on HC3 robust standard errors. *P* values were adjusted across the 19 cytokines using the Benjamini–Hochberg method.
