# Supplementary material for: Sex-Specific Cytokine Profiles Associated with Viral Load and Disease Severity in Patients with Severe Fever with Thrombocytopenia Syndrome
Source: Microorganisms. 2026 Oct 3;14(10):2242. doi: 10.3390/microorganisms14102242 (PMC13707548; doi:10.3390/microorganisms14102242)
Supplement: Supplementary file 1 [file microorganisms-14-02242-s001.zip › Supplemental Table S6.pdf]

Supplementary Table S6. Comparison of Spearman correlation coefficients between male and female patients

Clinical indicator	Cytokine	ρ (Male)	ρ (Female)	Δ ρ (Male − Female)	95% CI	*p* value	BH-adjusted *p* value
viral load	CCL4	0.614	0.388	0.226	(-0.11,0.561)	0.216	0.661
viral load	FGF2	0.432	0.422	0.009	(-0.362,0.373)	0.953	0.953
viral load	GM-CSF	0.353	0.443	-0.090	(-0.482,0.289)	0.632	0.953
viral load	IL-17	-0.193	-0.173	-0.020	(-0.422,0.382)	0.925	0.953
viral load	IL-8	0.382	0.061	0.321	(-0.102,0.726)	0.128	0.640
viral load	CXCL10	0.719	0.677	0.042	(-0.204,0.292)	0.763	0.953
viral load	IL-5	-0.016	-0.280	0.264	(-0.139,0.647)	0.176	0.661
viral load	PDGF-AB	0.003	-0.197	0.200	(-0.202,0.581)	0.313	0.661
viral load	TNF-α	0.620	0.329	0.290	(-0.079,0.645)	0.135	0.640
viral load	IFN-γ	0.597	0.608	-0.011	(-0.312,0.286)	0.941	0.953
viral load	IL-7	0.024	-0.012	0.036	(-0.394,0.46)	0.869	0.953
viral load	IL-4	0.316	0.105	0.211	(-0.19,0.595)	0.290	0.661
viral load	IL-6	0.369	0.415	-0.046	(-0.441,0.364)	0.829	0.953
viral load	IL-1RA	0.627	0.322	0.305	(-0.043,0.641)	0.072	0.640
viral load	CCL2	0.468	0.435	0.033	(-0.317,0.389)	0.862	0.953
viral load	CXCL9	0.639	0.447	0.192	(-0.168,0.550)	0.264	0.661
viral load	CCL11	0.353	0.301	0.051	(-0.32,0.427)	0.789	0.953
viral load	G-CSF	0.354	0.216	0.139	(-0.252,0.516)	0.484	0.919
viral load	IL-2	0.266	-0.201	0.467	(0.029,0.863)	0.038	0.640
AST	CCL4	0.621	0.427	0.194	(-0.128,0.511)	0.230	0.520
AST	FGF2	0.590	0.409	0.181	(-0.148,0.497)	0.246	0.520
AST	GM-CSF	0.344	0.417	-0.074	(-0.489,0.332)	0.718	0.747
AST	IL-17	-0.437	0.010	-0.448	(-0.798, -0.079)	0.014	0.264
AST	IL-8	0.529	0.241	0.288	(-0.086,0.643)	0.100	0.411
AST	CXCL10	0.578	0.439	0.140	(-0.157,0.450)	0.387	0.568
AST	IL-5	-0.191	-0.126	-0.065	(-0.467,0.346)	0.747	0.747
AST	PDGF-AB	0.147	-0.167	0.314	(-0.095,0.696)	0.108	0.411
AST	TNF-α	0.628	0.303	0.326	(-0.015,0.654)	0.056	0.355
AST	IFN-γ	0.525	0.287	0.238	(-0.117,0.593)	0.182	0.520
AST	IL-7	0.249	0.175	0.074	(-0.298,0.436)	0.695	0.747
AST	IL-4	0.309	0.216	0.093	(-0.302,0.482)	0.643	0.747
AST	IL-6	0.533	0.383	0.150	(-0.21,0.503)	0.388	0.568
AST	IL-1RA	0.624	0.275	0.350	(0.015,0.674)	0.038	0.355
AST	CCL2	0.558	0.343	0.216	(-0.127,0.554)	0.214	0.520
AST	CXCL9	0.583	0.431	0.152	(-0.187,0.488)	0.346	0.568
AST	CCL11	0.444	0.250	0.193	(-0.17,0.562)	0.304	0.568
AST	G-CSF	0.314	0.233	0.081	(-0.316,0.463)	0.695	0.747
AST	IL-2	0.159	0.066	0.093	(-0.324,0.475)	0.644	0.747
ALT	CCL4	0.469	0.332	0.137	(-0.234,0.512)	0.470	0.828
ALT	FGF2	0.472	0.361	0.111	(-0.253,0.469)	0.546	0.828
ALT	GM-CSF	0.309	0.237	0.072	(-0.355,0.491)	0.735	0.828
ALT	IL-17	-0.436	-0.045	-0.390	(-0.74,-0.006)	0.034	0.506

ALT	IL-8	0.446	0.074	0.371	(-0.026,0.753)	0.065	0.506
ALT	CXCL10	0.431	0.350	0.081	(-0.28,0.446)	0.656	0.828
ALT	IL-5	-0.278	-0.001	-0.276	(-0.663,0.144)	0.181	0.687
ALT	PDGF-AB	0.116	0.055	0.062	(-0.323,0.446)	0.741	0.828
ALT	TNF-α	0.518	0.200	0.318	(-0.033,0.668)	0.080	0.506
ALT	IFN-γ	0.391	0.236	0.156	(-0.225,0.54)	0.423	0.828
ALT	IL-7	0.286	0.267	0.020	(-0.339,0.374)	0.913	0.915
ALT	IL-4	0.225	0.205	0.020	(-0.364,0.401)	0.915	0.915
ALT	IL-6	0.372	0.203	0.169	(-0.246,0.564)	0.409	0.828
ALT	IL-1RA	0.487	0.217	0.270	(-0.122,0.649)	0.170	0.687
ALT	CCL2	0.424	0.208	0.215	(-0.196,0.621)	0.291	0.828
ALT	CXCL9	0.453	0.361	0.092	(-0.243,0.426)	0.587	0.828
ALT	CCL11	0.338	0.249	0.089	(-0.298,0.485)	0.645	0.828
ALT	G-CSF	0.223	0.102	0.121	(-0.29,0.512)	0.559	0.828
ALT	IL-2	0.204	0.034	0.170	(-0.247,0.544)	0.402	0.828
LDH	CCL4	0.633	0.320	0.313	(-0.084,0.702)	0.120	0.363
LDH	FGF2	0.668	0.412	0.257	(-0.066,0.59)	0.133	0.363
LDH	GM-CSF	0.349	0.353	-0.004	(-0.392,0.384)	0.983	0.983
LDH	IL-17	-0.359	-0.175	-0.184	(-0.534,0.172)	0.327	0.518
LDH	IL-8	0.501	0.178	0.323	(-0.086,0.707)	0.116	0.363
LDH	CXCL10	0.632	0.532	0.100	(-0.222,0.424)	0.530	0.630
LDH	IL-5	-0.100	-0.222	0.123	(-0.214,0.457)	0.502	0.630
LDH	PDGF-AB	0.105	-0.389	0.494	(0.108,0.838)	0.012	0.222
LDH	TNF-α	0.581	0.292	0.289	(-0.12,0.669)	0.152	0.363
LDH	IFN-γ	0.581	0.396	0.185	(-0.153,0.527)	0.290	0.501
LDH	IL-7	0.185	-0.047	0.232	(-0.181,0.634)	0.283	0.501
LDH	IL-4	0.394	0.086	0.308	(-0.087,0.693)	0.124	0.363
LDH	IL-6	0.545	0.438	0.107	(-0.267,0.478)	0.578	0.646
LDH	IL-1RA	0.643	0.288	0.355	(0.006,0.697)	0.043	0.363
LDH	CCL2	0.588	0.314	0.274	(-0.101,0.645)	0.172	0.363
LDH	CXCL9	0.607	0.472	0.135	(-0.179,0.467)	0.394	0.575
LDH	CCL11	0.442	0.144	0.298	(-0.123,0.706)	0.166	0.363
LDH	G-CSF	0.250	0.111	0.139	(-0.298,0.564)	0.523	0.630
LDH	IL-2	0.122	0.034	0.088	(-0.344,0.499)	0.683	0.721
CK	CCL4	0.525	0.279	0.247	(-0.125,0.608)	0.224	0.919
CK	FGF2	0.462	0.321	0.141	(-0.237,0.512)	0.470	0.919
CK	GM-CSF	0.160	0.264	-0.104	(-0.504,0.306)	0.617	0.919
CK	IL-17	-0.383	-0.229	-0.154	(-0.508,0.215)	0.418	0.919
CK	IL-8	0.336	0.209	0.128	(-0.284,0.526)	0.551	0.919
CK	CXCL10	0.434	0.390	0.044	(-0.307,0.402)	0.796	0.919
CK	IL-5	-0.135	-0.115	-0.020	(-0.413,0.386)	0.920	0.941
CK	PDGF-AB	0.127	-0.282	0.408	(0.014,0.764)	0.039	0.739
CK	TNF-α	0.451	0.212	0.239	(-0.128,0.597)	0.220	0.919
CK	IFN-γ	0.405	0.420	-0.015	(-0.378,0.368)	0.941	0.941

CK	IL-7	0.097	0.145	-0.048	(-0.457,0.354)	0.823	0.919
CK	IL-4	0.206	-0.017	0.223	(-0.195,0.616)	0.272	0.919
CK	IL-6	0.435	0.492	-0.057	(-0.405,0.304)	0.768	0.919
CK	IL-1RA	0.455	0.306	0.150	(-0.219,0.508)	0.400	0.919
CK	CCL2	0.479	0.330	0.149	(-0.218,0.506)	0.413	0.919
CK	CXCL9	0.384	0.302	0.082	(-0.315,0.464)	0.679	0.919
CK	CCL11	0.307	0.257	0.050	(-0.347,0.449)	0.810	0.919
CK	G-CSF	0.016	0.208	-0.191	(-0.572,0.197)	0.318	0.919
CK	IL-2	0.036	0.126	-0.091	(-0.496,0.314)	0.658	0.919
Platelet	CCL4	-0.512	-0.326	-0.186	(-0.538,0.173)	0.318	0.892
Platelet	FGF2	-0.262	-0.125	-0.137	(-0.521,0.25)	0.494	0.892
Platelet	GM-CSF	-0.212	-0.309	0.097	(-0.275,0.468)	0.613	0.892
Platelet	IL-17	-0.114	0.043	-0.157	(-0.563,0.262)	0.478	0.892
Platelet	IL-8	-0.230	-0.164	-0.067	(-0.478,0.346)	0.757	0.892
Platelet	CXCL10	-0.490	-0.429	-0.061	(-0.399,0.278)	0.721	0.892
Platelet	IL-5	0.021	0.020	0.001	(-0.381,0.383)	0.998	0.998
Platelet	PDGF-AB	0.050	0.462	-0.412	(-0.786,-0.018)	0.031	0.595
Platelet	TNF-α	-0.438	-0.386	-0.051	(-0.42,0.314)	0.798	0.892
Platelet	IFN-γ	-0.388	-0.201	-0.187	(-0.553,0.201)	0.346	0.892
Platelet	IL-7	0.109	-0.004	0.113	(-0.289,0.509)	0.566	0.892
Platelet	IL-4	-0.158	-0.325	0.167	(-0.217,0.554)	0.411	0.892
Platelet	IL-6	-0.297	-0.382	0.084	(-0.308,0.473)	0.659	0.892
Platelet	IL-1RA	-0.394	-0.202	-0.193	(-0.567,0.195)	0.326	0.892
Platelet	CCL2	-0.206	-0.289	0.083	(-0.32,0.483)	0.681	0.892
Platelet	CXCL9	-0.550	-0.466	-0.084	(-0.397,0.219)	0.576	0.892
Platelet	CCL11	-0.298	0.034	-0.332	(-0.697,0.055)	0.096	0.892
Platelet	G-CSF	-0.181	-0.165	-0.017	(-0.449,0.408)	0.943	0.995
Platelet	IL-2	-0.150	-0.056	-0.094	(-0.472,0.291)	0.619	0.892
WBC count	CCL4	0.005	-0.364	0.369	(-0.057,0.773)	0.074	0.260
WBC count	FGF2	-0.111	-0.143	0.032	(-0.369,0.44)	0.870	0.870
WBC count	GM-CSF	0.053	-0.215	0.268	(-0.143,0.663)	0.187	0.338
WBC count	IL-17	0.100	0.003	0.097	(-0.342,0.514)	0.666	0.703
WBC count	IL-8	-0.203	-0.085	-0.118	(-0.522,0.3)	0.568	0.654
WBC count	CXCL10	0.045	-0.250	0.295	(-0.127,0.702)	0.158	0.334
WBC count	IL-5	0.197	0.078	0.119	(-0.285,0.504)	0.586	0.654
WBC count	PDGF-AB	0.365	0.073	0.291	(-0.071,0.629)	0.122	0.289
WBC count	TNF-α	0.047	-0.217	0.264	(-0.169,0.686)	0.222	0.352
WBC count	IFN-γ	0.123	-0.316	0.440	(0.002,0.834)	0.043	0.204
WBC count	IL-7	0.232	-0.236	0.468	(0.058,0.837)	0.023	0.146
WBC count	IL-4	0.192	-0.145	0.337	(-0.07,0.722)	0.112	0.289
WBC count	IL-6	0.035	-0.197	0.231	(-0.187,0.622)	0.264	0.386
WBC count	IL-1RA	0.058	-0.294	0.352	(-0.056,0.753)	0.082	0.260
WBC count	CCL2	0.089	-0.393	0.482	(0.071,0.853)	0.015	0.143
WBC count	CXCL9	0.004	-0.172	0.175	(-0.264,0.602)	0.404	0.549

WBC count	CCL11	0.192	-0.322	0.514	(0.115,0.892)	0.013	0.143
WBC count	G-CSF	-0.132	-0.377	0.245	(-0.135,0.603)	0.196	0.338
WBC count	IL-2	0.030	-0.108	0.138	(-0.287,0.544)	0.522	0.654

Note: Δρ = ρ(Male) − ρ(Female). *P* values were adjusted for multiple comparisons using the Benjamini–Hochberg (BH) method.
