# Supplementary material for: Sex-Specific Cytokine Profiles Associated with Viral Load and Disease Severity in Patients with Severe Fever with Thrombocytopenia Syndrome
Source: Microorganisms. 2026 Oct 3;14(10):2242. doi: 10.3390/microorganisms14102242 (PMC13707548; doi:10.3390/microorganisms14102242)
Supplement: Supplementary file 1 [file microorganisms-14-02242-s001.zip › Supplemental Table S7.pdf]

Table S7. Sex × viral load interaction analyses for normalized cytokine changes in patients with SFTS.

Cytokine	N	Interaction Estimate	95% CI	*P* value	BH-adjusted *P* value
TNF-α	100	0.209	(-0.198, 0.616)	0.310	0.515
IL-6	100	0.050	(-0.498, 0.599)	0.856	0.856
IL-8	100	0.301	(-0.030, 0.633)	0.074	0.380
IL-7	100	0.013	(-0.052, 0.077)	0.692	0.767
CXCL10	100	0.188	(-0.053, 0.429)	0.124	0.380
CCL2	100	0.159	(-0.120, 0.437)	0.260	0.515
IFN-γ	100	0.132	(-0.342, 0.605)	0.583	0.738
IL-1RA	100	0.334	(0.108, 0.559)	0.004	0.078
PDGF-AB	100	0.038	(-0.029, 0.104)	0.266	0.515
CCL4	100	0.139	(-0.113, 0.391)	0.277	0.515
IL-4	100	0.120	(-0.035, 0.275)	0.127	0.380
IL-17	100	0.117	(-0.119, 0.353)	0.328	0.515
IL-2	100	0.259	(0.001, 0.517)	0.049	0.380
GM-CSF	100	0.032	(-0.142, 0.206)	0.716	0.767
FGF2	100	0.035	(-0.162, 0.232)	0.727	0.767
CXCL9	100	0.279	(-0.068, 0.625)	0.114	0.380
IL-5	100	0.060	(-0.020, 0.141)	0.140	0.380
G-CSF	100	0.297	(-0.334, 0.929)	0.352	0.515
CCL11	100	0.064	(-0.123, 0.252)	0.497	0.675

Note: Interaction estimates represent the difference in the association between viral load and normalized cytokine changes between males and females (male—female), with female sex as the reference category. Statistical inference was based on HC3 robust standard errors. *P* values were adjusted across the 19 cytokines using the Benjamini–Hochberg method.
