# Supplementary material for: Sex-Specific Cytokine Profiles Associated with Viral Load and Disease Severity in Patients with Severe Fever with Thrombocytopenia Syndrome
Source: Microorganisms. 2026 Oct 3;14(10):2242. doi: 10.3390/microorganisms14102242 (PMC13707548; doi:10.3390/microorganisms14102242)
Supplement: Supplementary file 1 [file microorganisms-14-02242-s001.zip › Supplemental Table S8.pdf]

Supplementary Table S8. Sex-stratified Spearman correlations between normalized cytokine ratios and laboratory indicators in patients with SFTS.

Sex	Laboratory indicator	Cytokine	Spearman's ρ	*P* value	BH-adjusted *P*
Male	AST	CCL4	0.621	<0.001	<0.001
Male	AST	FGF2	0.590	<0.001	<0.001
Male	AST	GM-CSF	0.344	0.015	0.021
Male	AST	IL-17	-0.437	0.001	0.002
Male	AST	IL-8	0.529	<0.001	<0.001
Male	AST	CXCL10	0.578	<0.001	<0.001
Male	AST	IL-5	-0.191	0.184	0.205
Male	AST	PDGF-AB	0.147	0.308	0.308
Male	AST	TNF-a	0.628	<0.001	<0.001
Male	AST	IFN-r	0.525	<0.001	<0.001
Male	AST	IL-7	0.249	0.081	0.096
Male	AST	IL-4	0.309	0.029	0.037
Male	AST	IL-6	0.533	<0.001	<0.001
Male	AST	IL-1RA	0.624	<0.001	<0.001
Male	AST	CCL2	0.558	<0.001	<0.001
Male	AST	CXCL9	0.583	<0.001	<0.001
Male	AST	CCL11	0.444	0.001	0.002
Male	AST	G-CSF	0.314	0.026	0.036
Male	AST	IL-2	0.159	0.271	0.286
Female	AST	CCL4	0.427	0.002	0.012
Female	AST	FGF2	0.409	0.003	0.012
Female	AST	GM-CSF	0.417	0.003	0.012
Female	AST	IL-17	0.010	0.944	0.944
Female	AST	IL-8	0.241	0.091	0.145
Female	AST	CXCL10	0.439	0.001	0.012
Female	AST	IL-5	-0.126	0.382	0.427
Female	AST	PDGF-AB	-0.167	0.247	0.293
Female	AST	TNF-a	0.303	0.033	0.077
Female	AST	IFN-r	0.287	0.044	0.092
Female	AST	IL-7	0.175	0.223	0.283
Female	AST	IL-4	0.216	0.131	0.178
Female	AST	IL-6	0.383	0.006	0.019
Female	AST	IL-1RA	0.275	0.053	0.101
Female	AST	CCL2	0.343	0.015	0.040
Female	AST	CXCL9	0.431	0.002	0.012
Female	AST	CCL11	0.250	0.079	0.137
Female	AST	G-CSF	0.233	0.104	0.152
Female	AST	IL-2	0.066	0.649	0.685
Male	ALT	CCL4	0.469	0.001	0.003
Male	ALT	FGF2	0.472	0.001	0.003

Male	ALT	GM-CSF	0.309	0.029	0.042
Male	ALT	IL-17	-0.436	0.002	0.004
Male	ALT	IL-8	0.446	0.001	0.004
Male	ALT	CXCL10	0.431	0.002	0.004
Male	ALT	IL-5	-0.278	0.051	0.064
Male	ALT	PDGF-AB	0.116	0.421	0.421
Male	ALT	TNF-a	0.518	<0.001	0.002
Male	ALT	IFN-r	0.391	0.005	0.009
Male	ALT	IL-7	0.286	0.044	0.060
Male	ALT	IL-4	0.225	0.116	0.133
Male	ALT	IL-6	0.372	0.008	0.013
Male	ALT	IL-1RA	0.487	<0.001	0.003
Male	ALT	CCL2	0.424	0.002	0.005
Male	ALT	CXCL9	0.453	0.001	0.004
Male	ALT	CCL11	0.338	0.016	0.026
Male	ALT	G-CSF	0.223	0.119	0.133
Male	ALT	IL-2	0.204	0.155	0.164
Female	ALT	CCL4	0.332	0.018	0.088
Female	ALT	FGF2	0.361	0.010	0.081
Female	ALT	GM-CSF	0.237	0.097	0.236
Female	ALT	IL-17	-0.045	0.755	0.844
Female	ALT	IL-8	0.074	0.608	0.770
Female	ALT	CXCL10	0.350	0.013	0.081
Female	ALT	IL-5	-0.001	0.992	0.992
Female	ALT	PDGF-AB	0.055	0.706	0.838
Female	ALT	TNF-a	0.200	0.163	0.238
Female	ALT	IFN-r	0.236	0.100	0.236
Female	ALT	IL-7	0.267	0.061	0.233
Female	ALT	IL-4	0.205	0.154	0.238
Female	ALT	IL-6	0.203	0.157	0.238
Female	ALT	IL-1RA	0.217	0.131	0.238
Female	ALT	CCL2	0.208	0.147	0.238
Female	ALT	CXCL9	0.361	0.010	0.081
Female	ALT	CCL11	0.249	0.082	0.236
Female	ALT	G-CSF	0.102	0.479	0.650
Female	ALT	IL-2	0.034	0.817	0.862
Male	LDH	CCL4	0.633	<0.001	<0.001
Male	LDH	FGF2	0.668	<0.001	<0.001
Male	LDH	GM-CSF	0.349	0.013	0.018
Male	LDH	IL-17	-0.359	0.010	0.015
Male	LDH	IL-8	0.501	<0.001	<0.001
Male	LDH	CXCL10	0.632	<0.001	<0.001
Male	LDH	IL-5	-0.100	0.490	0.490
Male	LDH	PDGF-AB	0.105	0.467	0.490

Male	LDH	TNF-a	0.581	<0.001	<0.001
Male	LDH	IFN-r	0.581	<0.001	<0.001
Male	LDH	IL-7	0.185	0.199	0.237
Male	LDH	IL-4	0.394	0.005	0.007
Male	LDH	IL-6	0.545	<0.001	<0.001
Male	LDH	IL-1RA	0.643	<0.001	<0.001
Male	LDH	CCL2	0.588	<0.001	<0.001
Male	LDH	CXCL9	0.607	<0.001	<0.001
Male	LDH	CCL11	0.442	0.001	0.002
Male	LDH	G-CSF	0.250	0.080	0.101
Male	LDH	IL-2	0.122	0.398	0.445
Female	LDH	CCL4	0.320	0.024	0.056
Female	LDH	FGF2	0.412	0.003	0.014
Female	LDH	GM-CSF	0.353	0.012	0.033
Female	LDH	IL-17	-0.175	0.225	0.305
Female	LDH	IL-8	0.178	0.217	0.305
Female	LDH	CXCL10	0.532	<0.001	0.001
Female	LDH	IL-5	-0.222	0.120	0.191
Female	LDH	PDGF-AB	-0.389	0.005	0.017
Female	LDH	TNF-a	0.292	0.040	0.073
Female	LDH	IFN-r	0.396	0.004	0.017
Female	LDH	IL-7	-0.047	0.744	0.785
Female	LDH	IL-4	0.086	0.552	0.617
Female	LDH	IL-6	0.438	0.001	0.009
Female	LDH	IL-1RA	0.288	0.042	0.073
Female	LDH	CCL2	0.314	0.026	0.056
Female	LDH	CXCL9	0.472	0.001	0.005
Female	LDH	CCL11	0.144	0.319	0.404
Female	LDH	G-CSF	0.111	0.442	0.525
Female	LDH	IL-2	0.034	0.813	0.813
Male	CK	CCL4	0.525	<0.001	0.002
Male	CK	FGF2	0.462	0.001	0.004
Male	CK	GM-CSF	0.160	0.268	0.364
Male	CK	IL-17	-0.383	0.006	0.011
Male	CK	IL-8	0.336	0.017	0.029
Male	CK	CXCL10	0.434	0.002	0.004
Male	CK	IL-5	-0.135	0.351	0.444
Male	CK	PDGF-AB	0.127	0.381	0.452
Male	CK	TNF-a	0.451	0.001	0.004
Male	CK	IFN-r	0.405	0.004	0.008
Male	CK	IL-7	0.097	0.502	0.561
Male	CK	IL-4	0.206	0.150	0.220
Male	CK	IL-6	0.435	0.002	0.004
Male	CK	IL-1RA	0.455	0.001	0.004

Male	CK	CCL2	0.479	<0.001	0.004
Male	CK	CXCL9	0.384	0.006	0.011
Male	CK	CCL11	0.307	0.030	0.047
Male	CK	G-CSF	0.016	0.910	0.910
Male	CK	IL-2	0.036	0.805	0.850
Female	CK	CCL4	0.279	0.050	0.106
Female	CK	FGF2	0.321	0.023	0.087
Female	CK	GM-CSF	0.264	0.064	0.122
Female	CK	IL-17	-0.229	0.109	0.173
Female	CK	IL-8	0.209	0.146	0.187
Female	CK	CXCL10	0.390	0.005	0.033
Female	CK	IL-5	-0.115	0.427	0.450
Female	CK	PDGF-AB	-0.282	0.047	0.106
Female	CK	TNF-a	0.212	0.139	0.187
Female	CK	IFN-r	0.420	0.002	0.023
Female	CK	IL-7	0.145	0.316	0.375
Female	CK	IL-4	-0.017	0.907	0.907
Female	CK	IL-6	0.492	<0.001	0.005
Female	CK	IL-1RA	0.306	0.031	0.090
Female	CK	CCL2	0.330	0.019	0.087
Female	CK	CXCL9	0.302	0.033	0.090
Female	CK	CCL11	0.257	0.071	0.123
Female	CK	G-CSF	0.208	0.148	0.187
Female	CK	IL-2	0.126	0.382	0.427
Male	Platelet	CCL4	-0.512	<0.001	0.001
Male	Platelet	FGF2	-0.262	0.066	0.139
Male	Platelet	GM-CSF	-0.212	0.140	0.240
Male	Platelet	IL-17	-0.114	0.432	0.505
Male	Platelet	IL-8	-0.230	0.108	0.205
Male	Platelet	CXCL10	-0.490	<0.001	0.002
Male	Platelet	IL-5	0.021	0.883	0.883
Male	Platelet	PDGF-AB	0.050	0.728	0.768
Male	Platelet	TNF-a	-0.438	0.001	0.007
Male	Platelet	IFN-r	-0.388	0.005	0.017
Male	Platelet	IL-7	0.109	0.452	0.505
Male	Platelet	IL-4	-0.158	0.274	0.372
Male	Platelet	IL-6	-0.297	0.036	0.085
Male	Platelet	IL-1RA	-0.394	0.005	0.017
Male	Platelet	CCL2	-0.206	0.152	0.240
Male	Platelet	CXCL9	-0.550	<0.001	0.001
Male	Platelet	CCL11	-0.298	0.035	0.085
Male	Platelet	G-CSF	-0.181	0.208	0.304
Male	Platelet	IL-2	-0.150	0.299	0.379
Female	Platelet	CCL4	-0.326	0.021	0.058

Female	Platelet	FGF2	-0.125	0.386	0.524
Female	Platelet	GM-CSF	-0.309	0.029	0.069
Female	Platelet	IL-17	0.043	0.764	0.908
Female	Platelet	IL-8	-0.164	0.256	0.374
Female	Platelet	CXCL10	-0.429	0.002	0.012
Female	Platelet	IL-5	0.020	0.888	0.937
Female	Platelet	PDGF-AB	0.462	0.001	0.007
Female	Platelet	TNF-a	-0.386	0.006	0.024
Female	Platelet	IFN-r	-0.201	0.161	0.279
Female	Platelet	IL-7	-0.004	0.977	0.977
Female	Platelet	IL-4	-0.325	0.021	0.058
Female	Platelet	IL-6	-0.382	0.006	0.024
Female	Platelet	IL-1RA	-0.202	0.161	0.279
Female	Platelet	CCL2	-0.289	0.042	0.088
Female	Platelet	CXCL9	-0.466	0.001	0.007
Female	Platelet	CCL11	0.034	0.816	0.912
Female	Platelet	G-CSF	-0.165	0.253	0.374
Female	Platelet	IL-2	-0.056	0.701	0.887
