# Supplementary material for: Sex-Specific Cytokine Profiles Associated with Viral Load and Disease Severity in Patients with Severe Fever with Thrombocytopenia Syndrome
Source: Microorganisms. 2026 Oct 3;14(10):2242. doi: 10.3390/microorganisms14102242 (PMC13707548; doi:10.3390/microorganisms14102242)
Supplement: Supplementary file 1 [file microorganisms-14-02242-s001.zip › Supplemental Table S9.pdf]

Supplementary Table S9. Adjusted associations between sex and normalized cytokine ratios in multivariable linear regression analyses.

Cytokine	N	β	95% CI	*P* value	BH-adjusted *P*
CCL4	100	-0.316	(-0.635, 0.003)	0.052	0.073
FGF2	100	-0.501	(-0.796, -0.206)	0.001	0.005
IL-17	100	-0.552	(-0.956, -0.148)	0.008	0.024
IL-8	100	-0.462	(-0.939, 0.014)	0.057	0.073
IL-5	100	0.357	(0.185, 0.529)	<0.001	<0.001
PDGF-AB	100	0.138	(0.012, 0.265)	0.033	0.059
CXCL10	100	0.465	(0.074, 0.855)	0.020	0.045
CCL11	100	0.130	(-0.173, 0.432)	0.396	0.396
IL-1RA	100	0.244	(-0.162, 0.650)	0.236	0.265

Note: Female sex was used as the reference group; negative β values indicate higher normalized cytokine ratios in females and positive values indicate higher ratios in males. *P* values were adjusted using the Benjamini–Hochberg (BH) procedure.
